# Multiple Genome Wide Association Mapping Models Identify Quantitative Trait Nucleotides for Brown Planthopper (*Nilaparvata lugens*) Resistance in MAGIC *Indica* Population of Rice

**DOI:** 10.3390/vaccines8040608

**Published:** 2020-10-14

**Authors:** Vanisri Satturu, Jhansi Lakshmi Vattikuti, Durga Sai J, Arvind Kumar, Rakesh Kumar Singh, Srinivas Prasad M, Hein Zaw, Mona Liza Jubay, Lakkakula Satish, Abhishek Rathore, Sreedhar Mulinti, Ishwarya Lakshmi VG, Abdul Fiyaz R., Animikha Chakraborty, Nepolean Thirunavukkarasu

**Affiliations:** 1 Institute of Biotechnology, Professor Jayashankar Telangana State Agricultural University, Rajendranagar, Hyderabad 500030, India; jdurgasai@gmail.com (D.S.J.); velamuriishwarya@gmail.com (I.L.V.); 2 Entomology, Pathology and Plant breeding Division, Indian Institute of Rice Research (ICAR-IIRR), Rajendranagar, Hyderabad 500030, India; jhansidrr@yahoo.co.in (J.L.V.); msprasad@gmail.com (S.P.M.); genefiyaz@gmail.com (A.F.R.); 3 Plant Breeding Division, International Rice Research Institute (IRRI)-South Asia Hub (SAH), Patancheru, Hyderabad 502324, India; a.kumar@irri.org; 4 Plant Breeding Division, International Rice Research Institute (IRRI), Metro Manila 1226, Philippines; r.singh@biosaline.org.ae (R.K.S.); heinzawagri@gmail.com (H.Z.); m.jubay@irri.org (M.L.J.); 5 Program Leader and Principal Scientist (Plant Breeding), Crop Diversification and Genetics, International Center for Biosaline Agriculture, Academic City, Dubai 14660, UAE; 6 Department of Agriculture, Plant Biotechnology Center, Shwe Nanthar, Mingalardon Township, Yangon 11021, Myanmar; 7 Department of Biotechnology Engineering, Ben-Gurion University of the Negev, Beer Sheva 84105, Israel; lsatish@post.bgu.ac.il; 8 Agriculture Statistics Division, International Crops Research for the Semi-Arid Tropics (ICRISAT), Patancheru, Hyderabad 502324, India; reach2abhi@gmail.com; 9 MFPI-Quality Control Lab, Professor Jayashankar Telangana State Agricultural University, Rajendranagar, Hyderabad 500030, India; mulisree1969@gmail.com; 10 Plant Breeding Division, Indian Institute of Millets Research (ICAR-IIMR), Rajendranagar, Hyderabad 500030, India; chakrabortyanimikha@gmail.com (A.C.); tnepolean@gmail.com (N.T.)

**Keywords:** association mapping, brown planthopper resistance, MAGIC, monophagous pest, QTNs, rice, SNPs

## Abstract

Brown planthopper (BPH), one of the most important pests of the rice (*Oryza sativa*) crop, becomes catastrophic under severe infestations and causes up to 60% yield loss. The highly disastrous BPH biotype in the Indian sub-continent is Biotype 4, which also known as the South Asian Biotype. Though many resistance genes were mapped until now, the utility of the resistance genes in the breeding programs is limited due to the breakdown of resistance and emergence of new biotypes. Hence, to identify the resistance genes for this economically important pest, we have used a multi-parent advanced generation intercross (MAGIC) panel consisting of 391 lines developed from eight *indica* founder parents. The panel was phenotyped at the controlled conditions for two consecutive years. A set of 27,041 cured polymorphic single nucleotide polymorphism (SNPs) and across-year phenotypic data were used for the identification of marker–trait associations. Genome-wide association analysis was performed to find out consistent associations by employing four single and two multi-locus models. Sixty-one SNPs were consistently detected by all six models. A set of 190 significant marker-associations identified by fixed and random model circulating probability unification (FarmCPU) were considered for searching resistance candidate genes. The highest number of annotated genes were found in chromosome 6 followed by 5 and 1. Ninety-two annotated genes identified across chromosomes of which 13 genes are associated BPH resistance including NB-ARC (nucleotide binding in APAF-1, R gene products, and CED-4) domain-containing protein, NHL repeat-containing protein, LRR containing protein, and WRKY70. The significant SNPs and resistant lines identified from our study could be used for an accelerated breeding program to develop new BPH resistant cultivars.

## 1. Introduction

Brown planthopper (BPH) (*Nilaparvata lugens* (Stål)), an outbreak-prone insect pest, causes heavy yield loss in rice (*Oryza sativa*) production ecologies. It has become a serious economic threat to rice production in several parts of Asia including India due to the rampant use of nitrogenous fertilizers and prophylactic insecticide applications. The famine death of one million people due to a severe attack of BPH was reported in Japan during 1732 [1]. The first and most severe outbreak of BPH with considerable economic damage in India was observed during 1973–1974, which had resulted in damage of almost 50,000 ha of rice crop [2,3]. Outbreaks of BPH were continued thereafter up to 1983 in different states and it attained number one pest status from 2007–2008 onwards due to its widespread outbreaks in India [4].

BPH is a monophagous pest. The mode of damage is a combination of two ways: 1. by laying egg masses in the midrib of the leaf sheath and blade thus blocking the xylem and phloem, and 2. continuously sucking sap from the phloem so that leaves become completely dry. This kind of symptom is commonly known as “hopper burn” since it visually creates a burning effect in the field but leads to total crop failure. All life forms—nymphs and adults—are capable of damaging the plant in all growth stages [5]. The infested plants are prone to secondary infection by sooty mold due to the excretion of honeydew by BPH. It is the carrier of grassy stunt and ragged stunt viral diseases [6].

Resistant rice cultivars with different stress tolerance systems reveal distinct resistant phenotypic characters to BPH, and consequently, its succession diversifies BPH populations [7]. So far, four known BPH biotypes were found [8,9] in rice ecologies. Biotype 1 belongs to East and Southeast Asia; Biotype 2, a dominant one, originated in Indonesia and Vietnam; Biotype 3 originated in the International Rice Research Institute (IRRI) laboratory [10]; and Biotype 4 is exclusive to the Indian subcontinent [11]. To date, about 38 *Bph*, 14 White-backed brown planthopper (WBph), 34 Small brown planthopper (SBph) resistance genes and about 100 quantitative trait locus (QTLs) were identified from *indica* sps., and other wild species of rice [11,12,13,14,15,16]. Though resistant cultivars have been developed and released for BPH such as IR26, IR36 and IR50, the evolution of new biotypes and breakdown of resistance are quite common in the improved cultivars. Resistance could be broken down when the resistance is governed by a single gene [17]. The heavy application of pesticides in rice cultivation also leads to pest resurgence, thus causing serious crop damage as well as the hazardous effects of pesticides on the grains. All these factors urged researchers to search for new resistant genetic and genomic resources for BPH.

Multi-parent advanced generation intercross (MAGIC) is developed by several generations of inter-mating of multiple founder parents [18]. Increased recombination events in the MAGIC developmental process result in increased mapping resolution and eliminate the effects of population sub-structures [19]. MAGIC has been successfully used to map QTLs in rice [20]. In rice, IRRI developed four MAGIC populations using sub-species-specific founders or a combination of founders, namely, MAGIC *indica*, *japonica* MAGIC, MAGIC *indica* plus, and Global MAGIC [21,22] to map multiple traits.

So far, no previous report is available for BPH resistance through the genome-wide association mapping (GWAS) approach in rice using high-density single nucleotide polymorphisms (SNPs). Here, we used a MAGIC *indica* population to identify the marker–trait associations through multiple GWAS models for BPH resistance using high-resolution SNP data. Six GWAS models were employed for the identification of significant SNPs and the results of the models were compared. The GWAS approach identified quantitative trait nucleotides (QTNs) associated with resistance genes and the results are discussed with reference to their utility in the rice breeding programmes.

## 2. Materials and Methods

### 2.1. Plant Material

**Figure 1:**
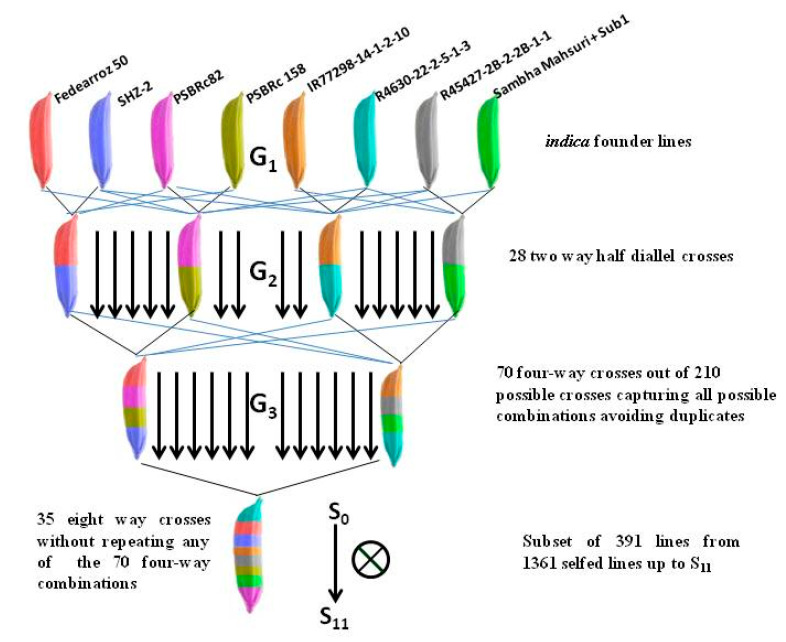
Development of the multi-parent advanced generation intercross *indica* (RiMi) population using eight *indica* founder lines.

The MAGIC *indica* population, hereafter called the RiMi panel, used in this study was developed at IRRI, Manila, Philippines [21]. The population development was initiated by crossing 8 elite *indica* founders, namely, Fedearroz 50, SANHUANGZHAN-2, IR64633-87-2-2-3-3 (PSBRc82), IR4630-22-2-5-1-3, IR45427-2B-2-2B-1-1, Samba Mahsuri +Sub1, IR77298-14-1-2-10 and IR77186-122-2-2-3 (PSBRc 158) in a half-diallel mating design. A set of 28 F1s were selected to develop 210 4-way crosses by avoiding duplicate crosses. The 4-way crosses were intermated two times and further selfed continuously until the S11 stage (Figure 1). A set of 1361 MAGIC lines were finally derived from the selfed population [22]. Of these, a sub-set of 391 randomly selected lines was used in the present experiment (Table S1).

### 2.2. Phenotyping, Scoring and Data Analysis

The Standard Seed box Screening Technique (SSST) [23] was used to screen the RiMi panel for BPH resistance in the glass-house facility at ICAR—the Indian Institute of Rice Research, Hyderabad, during the rainy seasons of 2016 and 2017 in an augmented complete block design. SSST is a rapid and efficient method to screen a large set of rice genotypes for the identification of field-resistant cultivars in the greenhouse [24,25]. A commonly used susceptible line, Taichung Native 1 (TN1), was used for the mass rearing of insects [10,23]. Adults were initially collected from the rice fields and the pure colony was maintained in the greenhouse at 30 °C ± 50 °C temperature, and 60 ± 5% relative humidity, which was congenial for the mass multiplication of BPH. The females were released on the 60-day-old potted plants placed in 70 × 75 cm oviposition cages. Plants with egg masses were selected and kept in different cages for nymphal emergence and the second to third instar nymphs were collected for infestation on the RiMi panel.

Around 15 sprouted seeds in each of the 391 RiMi panel lines were seeded in a standard seed tray with a size of 60L × 45W × 10H cm, which was filled with fertilizer-enriched puddled soil. In each tray, 20 MAGIC lines were sown by keeping two genotypes per row with an inter-line spacing of 3 cm. Susceptible check TN1 was covered in the borders and the resistant checks PTB33 and RP2068 were used in the middle rows of the seed tray. The trays with three-day-old seedlings were placed in big plastic trays containing water of 5 cm depth. The phenotyping was started by introducing six to eight second to third instar nymphs into 12 days-old seedlings [25]. The infestation was scored on a 0 to 9 scale [26] when 90% of the TN1 seedlings were succumbed. The augmented block design was conducted with two replications in 10 blocks containing 40 RiMi lines in all the blocks except in one with 31 RiMi lines per replication. Data on three checks (Two resistant and one susceptible) was recorded from every second tray considering the genotypes planted in two trays as one block. The material was evaluated in the augmented block design. The recorded data was subjected to analysis of variance using the R package “augmented RCBD” [27,28].

### 2.3. Genotyping and SNP Calling in GBS Pipeline

The MAGIC panel was genotyped by the Genotyping-By-Sequencing (GBS) approach [29] through Illumina HiSeq. The raw GBS data files (raw genotype files are available at http://snpseek.irri.org/_download.zul) were curated by GBS pipeline using Tassel 3.0.169 [30] by filtering at <30% missing and 0.05 minor allele frequency (MAF) [22]. A set of 27,041 SNPs identified in the subset of the 391 RiMi lines were considered for the present experiment.

### 2.4. Genome-Wide Association Analysis

The curated 27,041 SNPs and BPH phenotypic data of the RiMi panel were used for GWAS analysis using genetic association and prediction integrated tools (GAPIT) developed by [31]. GWAS was performed excluding founders, considering the MAGIC lines as unrelated individuals, since MAGIC population has a negligible population structure [21,32]. Four single-locus models such as the general linear model (GLM) [33], mixed linear model (MLM) [34,35], compressed mixed linear model (CMLM) [36] and settlement of MLM under progressively exclusive relationship (SUPER) [37], and two multi-locus models such as the multi locus mixed model (MLMM) [38] and fixed and random model circulating probability unification (FarmCPU) [36], were engaged to identify the marker–trait association. Bonferroni correction was applied to modify the threshold value and to control the false positive rate (FDR) in four single-locus GWAS. The results from all six models were compared to find out the consistency and repeatability of the associations. SNPs obtained by FarmCPU were considered for the candidate gene search for BPH resistance.

## 3. Results and Discussion

Plants are challenged by numerous herbivorous insects invade their natural environment. In consequence, they express diverse substantial and induced defense mechanisms, safeguarding themselves from herbivore attacks [39]. The first BPH resistant rice cultivar is IR26, followed by IR36 and IR50, which are improved by the IRRI. A number of QTNs against BPH in rice have been reported in this study. BPH is the most destructive monophagous, phloem sap-sucking insect, throughout Asia, causing significant yield losses to the rice crop [40,41]. BPH causes an annual estimated economic loss of more than 300 million USD in rice production ecologies of Asia [41,42].

So far, around 38 genes were mapped for BPH by several experiments through biparental mapping approaches. Almost 80% of the resistance genes were mapped in four major clusters on the rice genome using the mapping populations of *indica* and other wild species. Chromosome 4 has 12 genes that are clustered in three regions (*Bph30* and *Bph33* in a 0.91–0.97 Mb region between markers H99 and H101; *Bph3/17*, *Bph12*, *Bph15*, *Bph20(t)*, and *bph22(t)* in a 4.1–8.9 Mb region between markers RM8212 and B44; and *Bph6*, *bph18(t)*, *Bph27*, *Bph27(t)*, and *Bph34* in a 19.1–25.0 Mb region between markers RM16846 and RM6506), chromosome 12 L has eight genes (*Bph1*, *bph2*, *bph7*, *Bph9*, *Bph10*, *Bph18*, *Bph21* and *Bph26*) that are clustered together in a 19.1–24.4 Mb region between markers RM7102 and B122, five genes are on chromosome 6S (*Bph3/ Bph32bph4*, *Bph22*, *Bph25* and *bph29* ) in a 0.2–1.7 Mb region between markers S00310 and RM8101, and chromosome 3 has five genes (*Bph13* and *bph19(t)* on 3S [37], and *Bph11*, *Bph14*, and *Bph31* 3 L) [43,44,45,46,47,48].

Controlling the pest becomes ineffective since surface spray of pesticides could not reach the base of the infestation point. The rampant use of chemicals results in the development of resistance against many insecticides and the evolution of new biotypes. Recently, [41] reported that the excessive use of nitrogen fertilizers in the agricultural fields has an adverse impact on the environment, is valuable, and can advance the pest herbivores. The development of durable resistant varieties is not only an efficient measure to manage the pests, but also to conserve the ecological balance and safety. The BPH population in India, referred to as the South Asian biotype (Biotype 4), is more virulent than those in Southeast Asia (Biotype 2 and 3) [49,50]. Resistance genes identified for the Indian biotype are very few and are unable to offer broad-spectrum resistance. Here, a high-density SNP genotyped MAGIC population was used to improve the resolution of the trait association as well as to find the causal genes for resistance.

### 3.1. Response to Infestation

**Figure 2:**
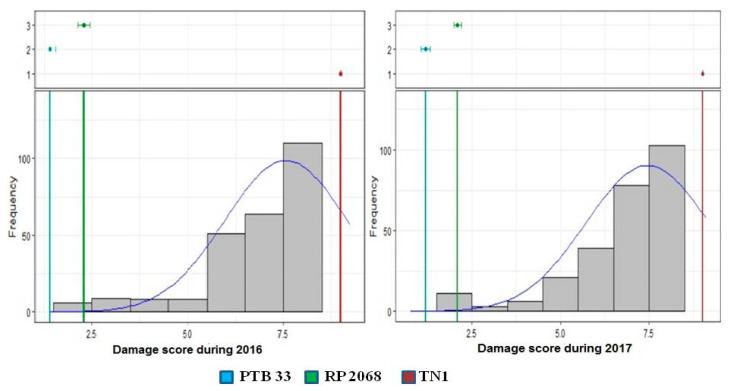
Histogram representation of frequency distribution of adjusted BPH scoring values of the RiMi panel. Top panel showing the performance of the checks as coloured lines in the histogram.

Screening at the seedling stage is an effective high-throughput method for the rapid and efficient screening of large germplasms as field screening offers inconsistent results due to improper control over the experimentation. A major difficulty in the screening of rice genotypes for BPH tolerance under field conditions is the lack of efficient screening techniques, problems associated with biotype selection, and environmental factors on the uniform density of the insect population. In SSST-based screening, a reliable phenotyping technique, phenotyping was done when all the TN1 (susceptible check) seedlings were dead. The infestation of the RiMI lines was scored on a 0 to 9 scale [26]. The scale of 0 to 3, 3 to 5, 5 to 7 and 7 to 9 were scored as resistant, moderately resistant, moderately susceptible, and susceptible, respectively. The results of the analysis of variance are shown in Table 1. The RiMi panel exhibited a broader variation for a phenotypic score in both the years. The ANOVA revealed a significant mean sum of squares for different sources of variation except for block (eliminating treatments). Frequency distribution-based adjusted mean values are represented in Figure 2. There was a significant skewness towards susceptibility, since the majority of the lines in the panel recorded a 7 to 9 score. This could be attributed to the fact that most of the RiMi lines were segregated for susceptibility. Twenty-three lines recorded a <5.0 score consistently in both of the seasons. TN1 showed complete susceptibility (9 score) whereas resistant checks PTB33 and RP2068 showed scores of less than two and three, respectively.

BPH resistance scores in both the years revealed a significant variation available for insect response in the RiMi panel (Table S2). Lines MIB_4393, MIB_3693, MIB_4077 and MIB_4570 were identified as the best lines for BPH resistance, since the score was less than two, which was equivalent to best check PTB33. Eight RiMi lines, MIB_4370, MIB_3850, MIB_4208, MIB_4241, MIB_4619, MIB_3409, MIB_4134 and MIB_4286, showed a similar response as that of the resistant check RP 2068. Eleven lines were found to be moderately resistant to BPH with the score of <5.0. Moderate susceptibility was recorded by 84 lines. A total of 191 and 93 RiMi lines showed susceptible (score 7) and highly susceptible (score 9) reaction towards BPH incidence.

Though 38 genes were reported for Biotype 4 from landraces and wild species [44,49,51], resistance was very low and donors were inferior in agronomic and quality traits. The popular BPH resistance sources, PTB33 (Bph2 and Bph3 donor) and Rathu Heenati (Bph3 and Bph17 donor), are agronomically inferior, hence identification of superior segregants with farmers’ preferred slender grain in the recombinant programme is always a challenge.

The 391 RiMi lines used in the study were selected for superior agronomic and grain quality traits with BPH resistance. Better combinations of segregants for these traits were achieved in the resultant population since MAGIC provides more rounds of recombination by its design and makes it possible to break genetic drag and leads to novel genetic rearrangements with greater genetic diversity [32,52,53].

Among the RiMi lines, the identified MIB_4393, MIB_3693, MIB_4077, and MIB_4570, which are similar to PTB33 in resistance, could be used as new sources of resistance for developing varieties without genetic drag in the breeding programmes.

### 3.2. Marker and Covariates

**Figure 3:**
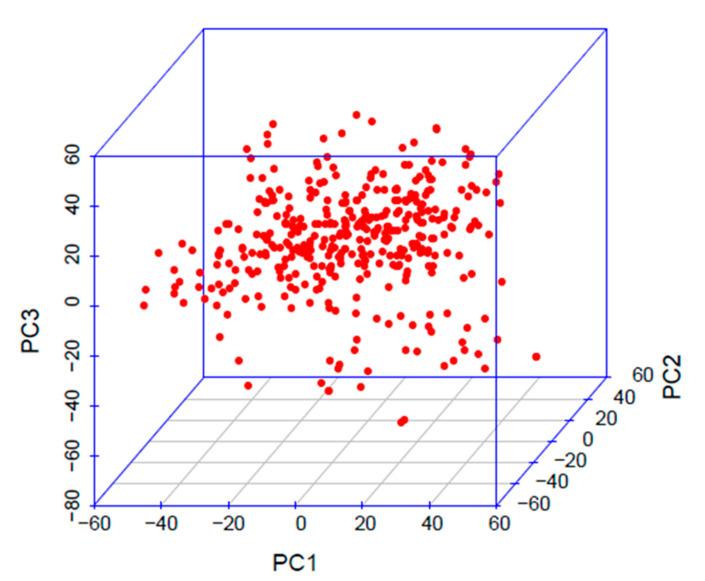
3D scatter plot of principal component analysis for single nucleotide polymorphisms data of the RiMi panel.

A total of 27,041 polymorphic SNPs developed in the RiMi panel were used in this experiment. The SNP set with the MAF = 0.05 and with <30% missing data, was distributed across 12 chromosomes, with an average of 2253 SNPs per chromosome. The highest number of SNPs was detected on chromosome 1 (3495 SNPs) and the lowest on chromosome 9 (1386 SNPs). The grouping pattern of the RiMi panel was inferred using principal component analysis (PCA). The RiMi panel explained the uniform distribution of alleles without any population structure (Figure 3). Principal components that explained up to 50% of the total variation were included in the GWAS models to correct any uneven allele frequencies. Kinship matrices built in the respective association mapping models were used for the additional correction of false positives.

### 3.3. Identification of SNPs

MAGIC populations developed in several crops including rice serve as potential genetic resources for the identification of QTLs. In the global MAGIC population of rice, 38 and 34 QTLs for yield and grain quality traits were identified through GWAS and interval mapping, respectively [20]. GWAS analysis was conducted using mixed linear models in the MAGIC *indica* plus population of rice, and 57 significant genomic regions for agronomic and bio-fortification traits were detected [54].

Here, we conducted the GWAS analysis in the MAGIC population using four single-locus models (GLM, MLM, CMLM and SUPER) and two multi-locus models (MLMM and Farm CPU) to identify the common SNPs associated with BPH resistance (Figure S1). Each model has its own characteristics in terms of statistical power, selection of covariates, grouping of markers, computational power, ability to control false positives and the ability to include true associations, and so on [55]. Considering these factors and the trait complexity, we wanted to engage all these models to find out the marker–trait association, and the results from the different models are compared.

We considered the *p* value <10^−3^ to identify significant SNPs across models and, using this threshold, a total of 248 associated SNPs that were significant for BPH resistance were detected for the mean BPH data. These SNPs were distributed on chromosomes 1, 2, 4, 5, 6, 7, 10, 11, and 12. The order of SNPs detected by various models is as follows: GLM (213), Farm CPU (190), MLMM (167), SUPER (162), MLM (91) and CMLM (80) (Table S3).

We have identified a maximum of 213 SNPs by employing the GLM model mapped on chromosomes 1, 2, 4, 5, 6, 7, 10 and 12. GLM detected the highest number of associations since it is the less stringent model among all, and hence, it is prone to detect more false positives. Both the MLM and CMLM methods identified the lowest number of SNPs (91 and 80, respectively) compared to other models. CMLM is an improvised MLM procedure by way of grouping the markers while a generating kinship relationship that could have reduced SNP associations over MLM. Among the single locus models used in this study, SUPER was the only method that could identify the marker–trait associations on chromosome 11 in addition to chromosomes 1, 2, 4, 5, 6, 7, 10, and 12. SUPER uses a subset of markers to compute the kinship, hence the statistical power is improved over MLM [37].

The MLMM is one of the multi-locus models, and it uses forward selection and backward elimination of SNPs based on heritable variance. This method identified 167 marker-trait associations that are distributed over chromosomes 1, 2, 4, 5, 6, and 10. Farm CPU is an improvised approach by combining the advantages of MLLM and SUPER to achieve better statistical power and computational efficiency [55]. A total of 190 SNPs were identified through FarmCPU, of which 190, 78, 69, 138, and 136 were co-mapped with GLM, MLM, CMLM, MLMM, and SUPER, respectively. In total, 58 common SNPs on chromosome 1 (7 SNPs), 2 (1 SNPs), 4 (1 SNPs), 5 (11 SNPs), 6 (36 SNPs) and 10 (2 SNPs) were mapped across models (Table 2).

**Figure 4:**
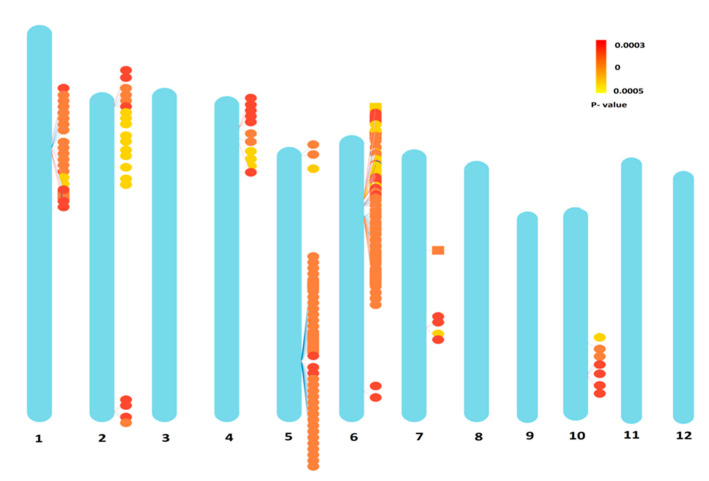
Distribution of significant SNPs on 12 rice chromosomes identified by FarmCPU.

**Figure 5:**
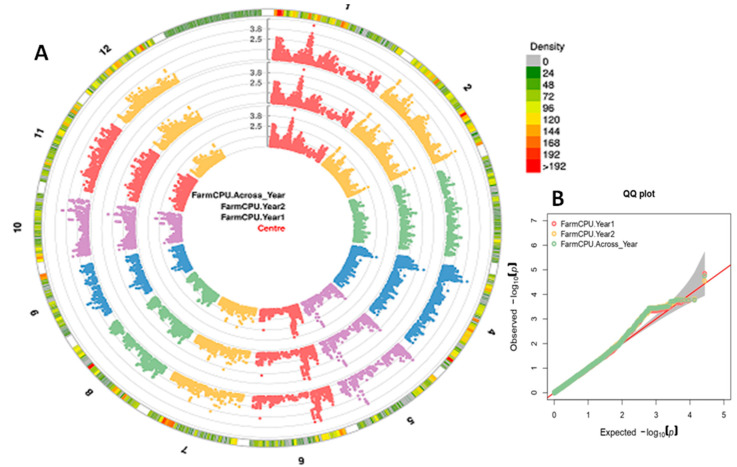
(**A**) Circular-Manhattan plot; (**B**) Quantile–quantile (QQ) plots of Farm CPU showing the significant SNP associations (*p* < 0.001) with BPH resistance for the two test years and across years. In the panel A, 1–12 indicate chromosome numbers.

Since FarmCPU is the comprehensive model among all, we considered the results obtained by FarmCPU for identification of resistance genes associated with the SNPs. The identified 190 significant SNP loci located on chromosomes 1 (20 SNPs), 2 (20 SNPs), 4 (11 SNPs), 5 (39 SNPs), 6 (88 SNPs), 7 (5 SNPs) and 10 (7 SNPs) were used for searching genes in their vicinity (Figure 4). The Manhattan plots and QQ plots for BPH resistance during 2016, 2017 and for the mean over years from FarmCPU are presented in Figure 5. Manhattan plots and QQ plots of GWAS for the other five models are submitted as Supplementary Figure S2.

Four important SNP clusters were identified on chromosome 1 and 2, where 20 SNPs were distributed in these regions (Table S3). One cluster was located in the 12 to 14.5 Mb region on the short arm of chromosome 1 and three clusters were located in the 1 to 2 Mb, 7.5 to 8.5 Mb and 34 to 35.5 Mb regions on both the short and long arms of chromosome 2. Significant SNPs identified on a long arm of chromosome 2 were mapped near (approximately 2 Mb regions away from) the *Bph13* (t) gene reported in *O. eichingeri* [56] (Figure 4).

Eleven significant SNPs that were identified at the 3 to 5.5 Mb region on the short arm of the chromosome 4 were also co-localized with Qbph4, *Bph17* [57] and *Bph12* [58]. Co-localization of the significant SNPs in the vicinity of the identified resistance gene locations such as *Bph13* explained the fact that the founder lines are directly or indirectly descended from any one of those wild species [59].

Two regions with 39 SNPs spanning a region 0.7 to 1.1 Mb (short arm) and 23.0 to 24.0 Mb (long arm) were identified on chromosome 5. It is to be noted that the long arm of chromosome 5 was earlier mapped for Qbph5 [60], indicating the importance of this region for searching resistance gene(s).

Chromosome 6 showed one major cluster of SNPs on short arm from 6.0 to 9.0 Mb with 86 SNPs. Bph32 was identified and located 4 Mb away from this region [61]. Five SNPs were clustered on chromosome 7 at a physical distance from 10 to 19.5 Mb and seven closely located SNPs mapped on the short arm of chromosome 10 from the 18 to 19 Mb region. Qbph7 [62] mapped on the long arm of chromosome 7 was found within the identified SNP cluster.

### 3.4. Identification for Candidate Genes for Resistance Based on SNPs

The genes associated with BPH resistance were extracted from the annotated gene search in the Rice Genome Annotation Project (Michigan State University Rice Genome Annotation Project (MSU-RAP) database (Osa1) Release 7), to identify the novel candidate genes. The results are given in Table S4. The search produced 92 annotated genes associated with 189 Quantitative Trait Nucleotides (QTNs). Chromosome 6 mapped the greatest number of genes (39) followed by chromosome 5 (18 genes) and chromosome 1 (12 genes). Among the 39 genes on chromosome 6, nine were found to have a defense-related functional mechanism (Table 3). Genes *LOC_Os06g11010*, *LOC_Os06g12160*, *LOC_Os06g12360*, *LOC_Os06g12610*, *LOC_Os06g12870*, *LOC_Os06g13600*, *LOC_Os06g14510*, *LOC_Os06g15750* and *LOC_Os06g15820* represent stress responsive proteins, eukaryotic aspartyl protease domain containing protein, AAA-type ATPase family protein, pentatricopeptide, auxin efflux carrier component, leaf senescence related protein, HEAT repeat family protein, glucose-6-phosphate isomerase, NB-ARC domain containing protein, and NHL repeat-containing protein, respectively. Twenty-one casual QTNs (S6_5759360, S6_6513819, S6_6514012, S6_6697070, S6_6869009, S6_7045328, S6_7045355, S6_7531094, S6_7531433, S6_7531437, S6_7538495, S6_8153078, S6_8932488, S6_8977107, S6_8977116, S6_8977156, S6_8977190, S6_8977712, S6_8977949, S6_8977972, and S6_8982135) were associated with these genes. A gene *LOC_Os06g18600* associated with S7_10995384 was also found to encode a stress responsive gene OsFBL37-F-box domain and LRR containing protein. *Bph9*, *Bph18* and *Bph26* on chromosome 12 [63,64,65] and *Bph14* on chromosome 3 [43] were identified to encode proteins, namely, coiled-coil, nucleotide-binding-site and leucine-rich repeat (CC-NBS-LRR).

On chromosome 5, eight significant QTNs, S5_23249078, S5_23249119, S5_23249125, S5_23249237, S5_23249605, S5_23310970, S5_23312204 and S5_23314218 are mapped in the genic locations of LOC_Os05g39590 and LOC_Os05g39720 encoding AP2 domain containing protein and WRKY70. These two transcription factors are involved in a stress tolerant mechanism that could contribute to BPH resistance. The AP2 (APETALA2)/EREBP (Ethylene-Responsive Element-Binding Protein) family has an important regulatory function in environmental stress tolerance, that regulates diverse processes of plant development and metabolism. It mainly regulates ABA dependent or ABA independent stress responsive pathways and is also known to be involved in biotic stress response through the Jasmonic acid pathway and in abiotic stress response through the ABA biosynthetic pathway [66]. WRKY, a class of TF, is involved in plant stress responses including biotic stresses [67]. A class of WRKY, known as *OsWRKY13,* mediated the gene regulation through Salicylate and Jasmonate-dependent signaling pathways in rice disease resistance [68]. The *Bph14* resistance gene is associated with WRKY46 and WRKY72 by protecting them from degradation, thus providing resistance [69]. The above-mentioned WRKY TFs provide BPF resistance by over-expressing the *RLCK281* and *callose synthase* genes.

A gene *LOC_Os01g24690* is associated with QTN S1_13898444 among 12 annotated genes identified on chromosome 1, encoding 60S ribosomal protein L23A. Ribosomal proteins have multiple functions and differentially regulate at the time of stress conditions [70]. The higher expression of ribosomal proteins is important for the proper functioning of several house-keeping genes during the stress conditions [71] and the QTN associated with such proteins could be useful for BPH resistance.

Allelic status of the significant QTNs that contribute to resistance in the agronomically superior RiMi lines (MIB_4393, MIB_3693, MIB_4077 and MIB_4570) are presented in Table 4. These lines could be used as new sources of resistance in the breeding programs as well as to explore downstream genes in genetic studies.

## 4. Conclusions

Over the past few years, substantial developments have been made in rice molecular breeding for high yield, biotic and abiotic stress tolerance, and grain quality, etc. Molecular breeding for BPH pest resistance in rice was, however, constrained because of the complexity of interrelatedness among BPH and rice. BPH is one of the most economically important insect pests of rice. Severe infestation in high-input rice ecologies results in major yield loss in susceptible varieties. Host-plant resistance is the best way to protect the environment and preserve the ecological balance of rice growing areas. The MAGIC panel identified 59 common SNPs on different rice chromosomes through six association mapping models. The gene search associated with QTNs revealed 13 stress tolerance genes that would play a significant role in BPH resistance. The 31 significant QTNs associated with the resistance genes could be used in accelerated marker-assisted breeding to develop BPH resistant varieties. Identified BPH resistant lines could be directly used in varietal release programs based on their agronomic performance and used as donors for BPH resistance to develop new resistant cultivars.

## Figures and Tables

**Table 1:** Analysis of variance and mean sum of squares of augmented block design for brown planthopper scoring of the 391RiMi panel.

Source	df	2016	2017	Mean Over Years
Treatment (ignoring blocks)	393	4.07 **	4.47 **	4.30 **
Treatment: Check	2	172.43 **	182.1 **	178.53 **
Treatment: Test vs. Check	1	313.6 0 **	318.46 **	318.45 **
Treatment: Test	390	2.42 **	2.76 **	2.60 **
Block (eliminating treatments)	9	0.08 ns	0.08 ns	0.09 ns
Residuals	18	0.21	0.10	0.09

** Significance at 1% probability level (*p* value = 0.001). df: degrees of freedom.

**Table 2:** Total number and Common Single Nucleotide Polymorphisms mapped across Genome-Wide Association Mapping models.

Chromosome	GLM	MLM	CMLM	SUPER	Farm CPU	MLMM	Common SNPs
1	28	12	8	30	20	26	S1_12742211, S1_13273091, S1_13274423, S1_13365703, S1_13374131, S1_13374151, S1_13424589
2	21	1	5	13	20	13	S2_34796411
4	13	2	1	11	11	4	S4_4571354
5	40	14	14	39	39	40	S5_23256042, S5_23257107, S5_23257108, S5_23279024, S5_23279042, S5_23287785, S5_23303726, S5_2330752, S5_23310970, S5_23312204, S5_23314218
6	90	49	40	60	88	61	S6_6769492, S6_6769526, S6_6769533, S6_7646336, S6_8850552, S6_8851568, S6_8870236, S6_8876540, S6_8876586, S6_8876606, S6_8876610, S6_8886408, S6_8886434, S6_8914643, S6_8914650, S6_8914651, S6_8914671, S6_8914769, S6_8917185, S6_8917933, S6_8917936, S6_8917962, S6_8921200, S6_8921201, S6_8932488, S6_8956202, S6_8962742, S6_8962796, S6_8975500, S6_8977107, S6_8977116, S6_8977156, S6_8977190, S6_9003844, S6_9003866, S6_9004225
7	6	-	-	1	5	-	-
10	8	9	8	2	7	19	S10_18127881, S10_18145180
11	-	-	-	2	-	-	-
12	7	4	4	4	-	4	-
Total	213	91	80	162	190	167	

**Table 3:** Quantitative Trait Nucleotides (QTNs) and associated genes identified with defense-responsive mechanisms.

S. No.	Gene locus ID	Chromosome	QTNs	Putative Function
1	LOC_Os01g24690	1	S1_13898444	60S ribosomal protein L23A, putative, expressed, response to abiotic stimulus, response to stress
2	LOC_Os05g39590	5	S5_23249078, S5_23249119, S5_23249125, S5_23249237, S5_23249605	AP2 domain containing protein, expressed
3	LOC_Os05g39720	5	S5_23310970, S5_23312204, S5_23314218	WRKY70, expressed
4	LOC_Os06g11010	6	S6_5759360	Eukaryotic aspartyl protease domain containing protein, expressed
5	LOC_Os06g12160.1	6	S6_6513819, S6_6514012	AAA-type ATPase family protein, putative, expressed
6	LOC_Os06g12360.1	6	S6_6697070	Pentatricopeptide, putative, expressed
7	LOC_Os06g12610.1	6	S6_6869009	Auxin efflux carrier component, putative, expressed
8	LOC_Os06g12870.1	6	S6_7045328, S6_7045355	Leaf senescence related protein, putative, expressed
9	LOC_Os06g13600.1	6	S6_7531094, S6_7531433, S6_7531437, S6_7538495	HEAT repeat family protein, putative, expressed
10	LOC_Os06g14510.3	6	S6_8153078	Glucose-6-phosphate isomerase, putative, expressed
11	LOC_Os06g15750	6	S6_8932488	NB-ARC domain containing protein, expressed
12	LOC_Os06g15820.1	6	S6_8977107, S6_8977116, S6_8977156, S6_8977190, S6_8977712, S6_8977949, S6_8977972, S6_8982135	NHL repeat-containing protein, putative, expressed
13	LOC_Os07g18600	7	S7_10995384	OsFBL37-F-box domain and LRR containing protein, expressed

**Table 4:** Allelic status of the significant QTNs that are contributing to resistance in the agronomically superior RiMi lines (MIB_4393, MIB_3693, MIB_4077 and MIB_4570).

S. No	Chromosome	SNP Position	Allelic Status	Putative Function
1	5	23249125	C	AP2 domain containing protein, expressed
2	6	5759360	T	Eukaryotic aspartyl protease domain containing protein, expressed
3	6	6513819	T	AAA-type ATPase family protein, putative, expressed
4	6	6514012	G	
5	6	6697070	T	Pentatricopeptide, putative, expressed
6	6	6869009	G	auxin efflux carrier component, putative, expressed
7	6	7045328	C	Leaf senescence related protein, putative, expressed
8	6	7045355	G	
9	6	7531094	C	HEAT repeat family protein, putative, expressed
10	6	7538495	G	
11	6	8153078	G	Glucose-6-phosphate isomerase, putative, expressed
12	7	10995384	C	OsFBL37 - F-box domain and LRR containing protein, expressed

## Supplementary Materials

The following are available online at https://www.mdpi.com/2076-393X/8/4/608/s1, Figure S1: Graphic representation of physical locations of the common SNPs for BPH resistance identified using single and multi-locus models on all chromosomes, Figure S2: Manhattan plots and Quantile–quantile (QQ) plots of different models for the two test years and across years, Table S1: Pedigree details of 391 R-MI population, Table S2: BPH resistance scores of 391 R-MI population across years, Table S3: SNP trait associations identified by different single and multi-locus GWAS models, Table S4: List of candidate genes (defense related genes) identified in the associated QTN regions.
