# Supplementary material for: Multiple Genome Wide Association Mapping Models Identify Quantitative Trait Nucleotides for Brown Planthopper (Nilaparvata lugens) Resistance in MAGIC Indica Population of Rice
Source: Vaccines (Basel). 2020 Oct 14;8(4):608. doi: 10.3390/vaccines8040608 (PMC7712083; doi:10.3390/vaccines8040608)
Supplement: Supplementary file 1 [file vaccines-08-00608-s001.zip › Supplementary figures 952340.pdf]

Supplementary Figures

Supplementary Figure S1: Graphic representation of physical locations of the common SNPs for BPH resistance identified using single and multi locus models on all chromosomes

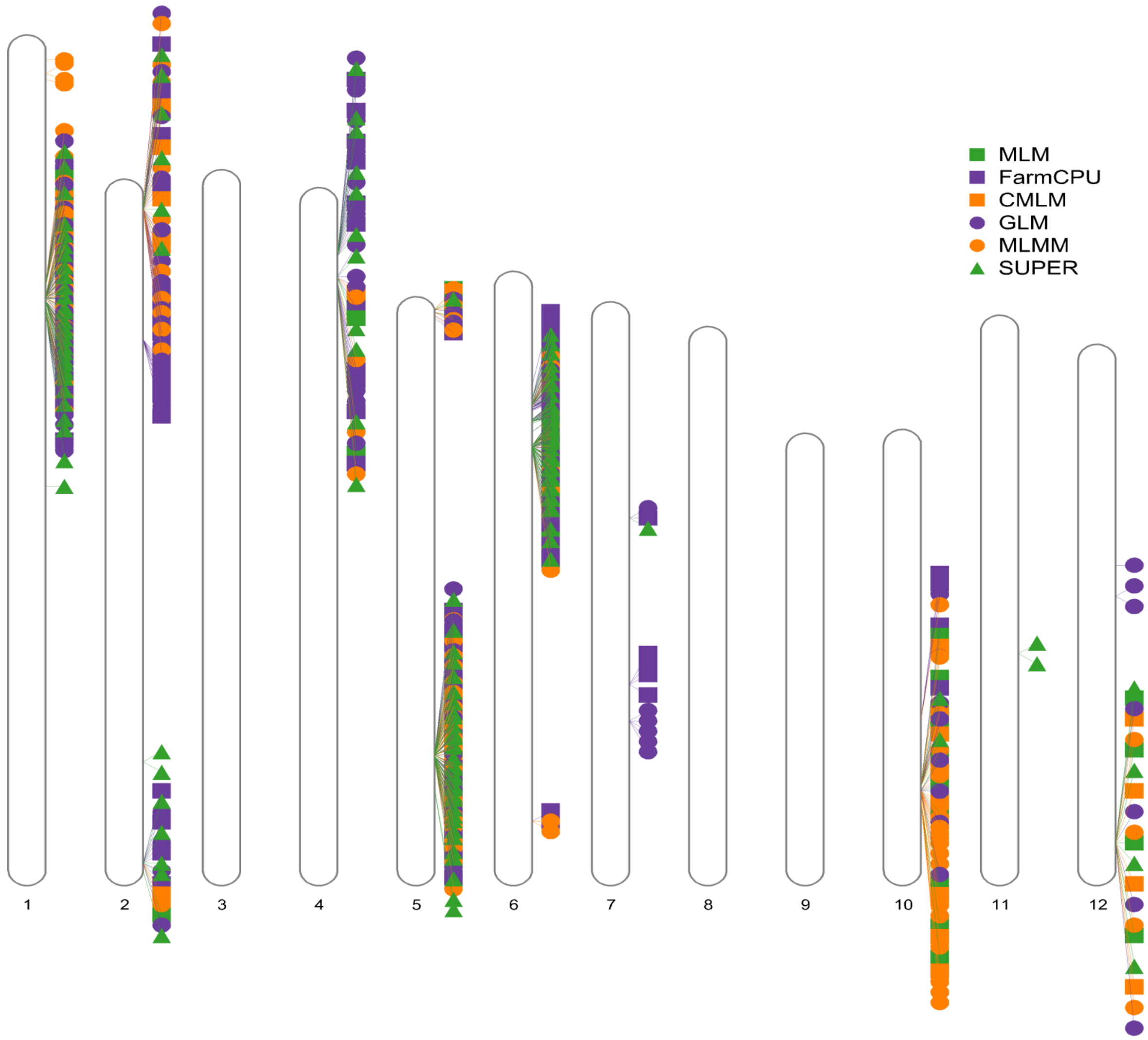

Supplementary Figure S2: Manhattan plots and Quantile-quantile (QQ) plots of different models for the two test years and across years

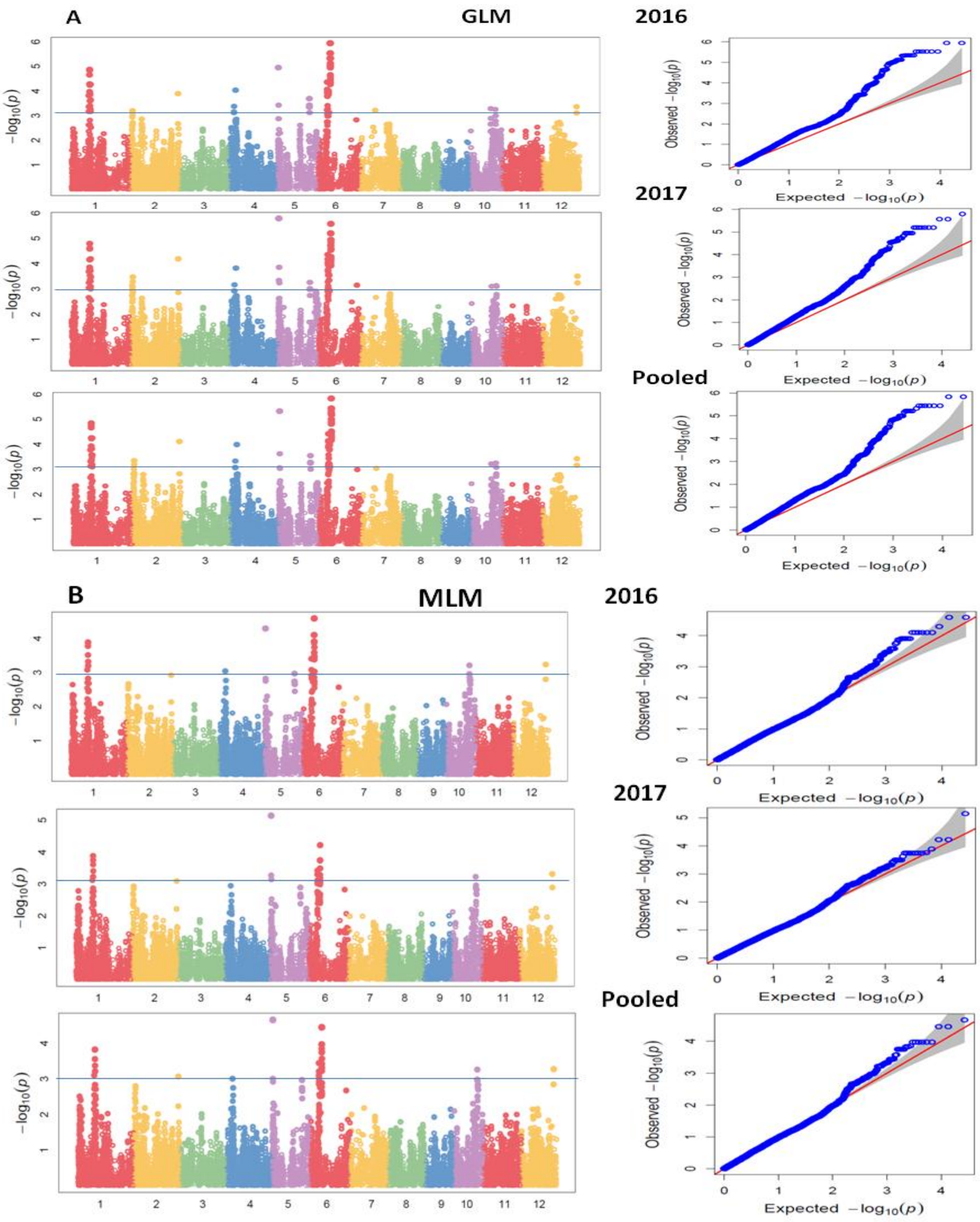

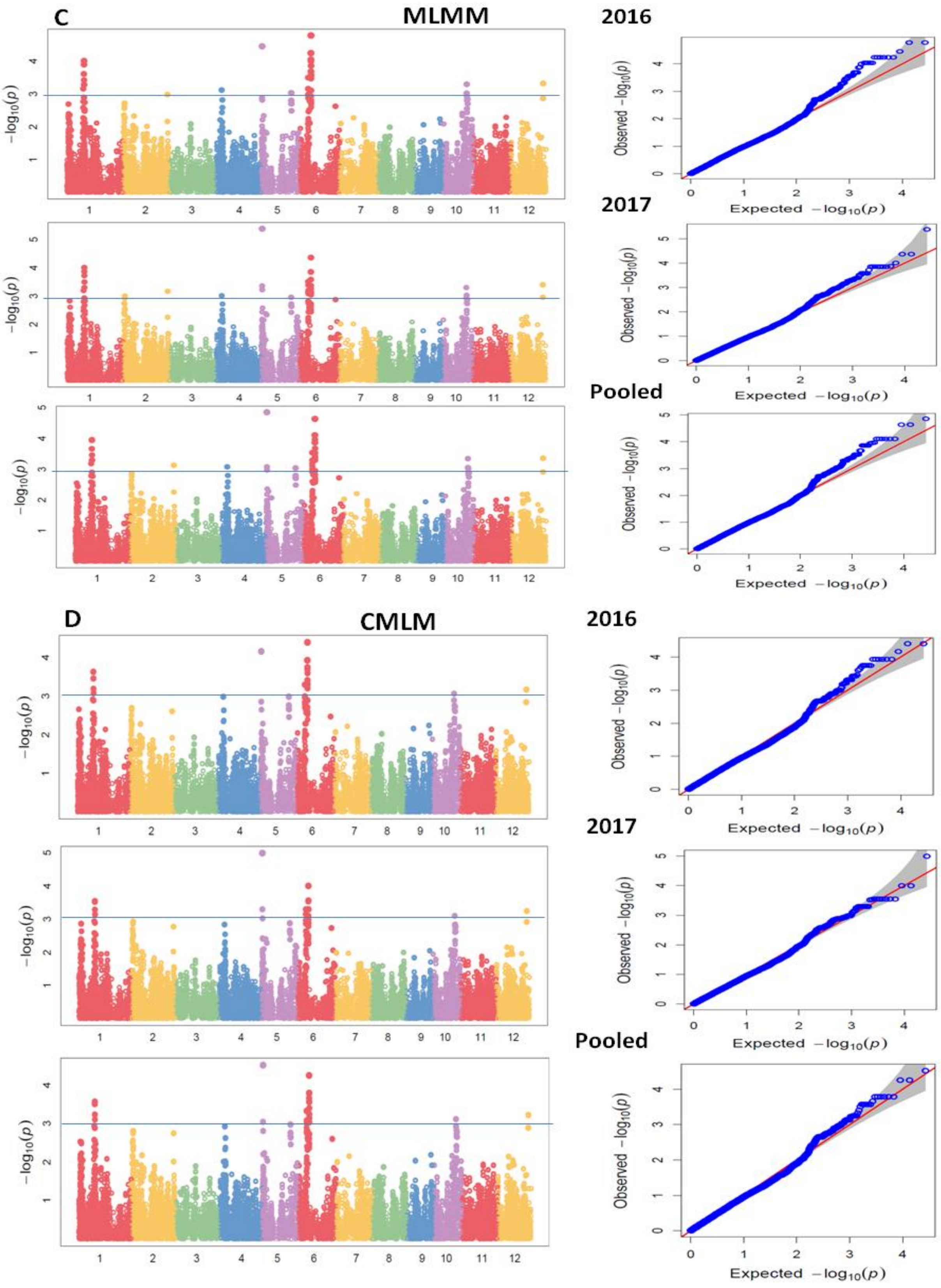

C
MLMM
2016
2017
Pooled
D
CMLM
2016
2017
Pooled
$-\log_{10}(p)$
Observed $-\log_{10}(p)$
Expected $-\log_{10}(p)$
1 2 3 4 5 6 7 8 9 10 11 12

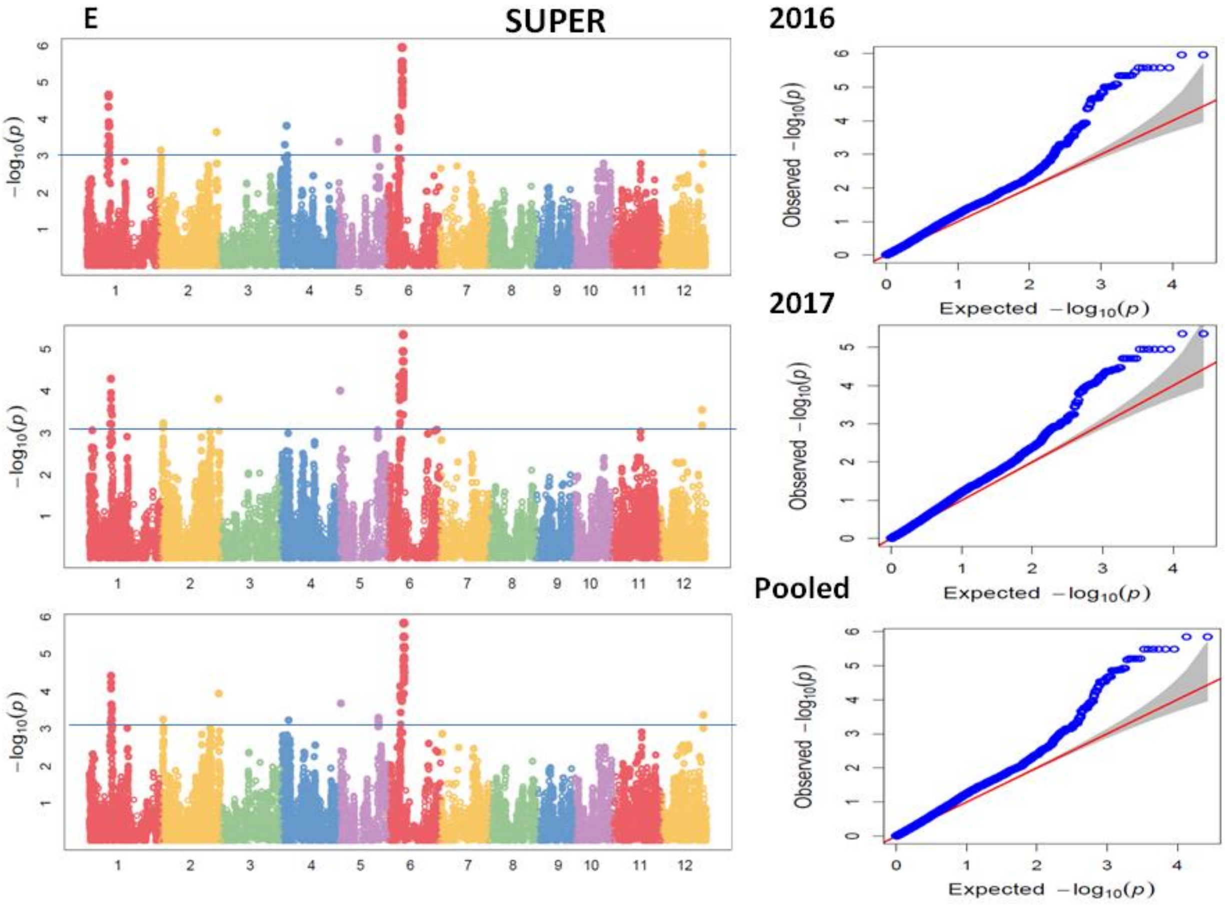

Figure S2 A) GLM

Figure S2 B) MLM

Figure S2 C) MLMM

Figure S2 D) CMLM

Figure S2 E) SUPER
