# Supplementary material for: Multiple Genome Wide Association Mapping Models Identify Quantitative Trait Nucleotides for Brown Planthopper (Nilaparvata lugens) Resistance in MAGIC Indica Population of Rice
Source: Vaccines (Basel). 2020 Oct 14;8(4):608. doi: 10.3390/vaccines8040608 (PMC7712083; doi:10.3390/vaccines8040608)
Supplement: Supplementary file 1 [file vaccines-08-00608-s001.zip › Supplementary Tables 952340.pdf]

Supplementary Table S1: Pedigree details of 391 RiMi population

S. NO.	GBS CODE	DESIGNATION
1	MIB_3400	IR 93326:9-B-21-3-4-1RGA-2RGA-1-B-B
2	MIB_3406	IR 93326:15-B-22-12-4-1RGA-2RGA-1-B-B
3	MIB_3408	IR 93326:17-B-8-5-7-1RGA-2RGA-1-B-B
4	MIB_3409	IR 93326:18-B-12-7-15-1RGA-2RGA-1-B-B
5	MIB_3414	IR 93326:23-B-4-8-12-1RGA-2RGA-1-B-B
6	MIB_3415	IR 93326:24-B-11-8-16-1RGA-2RGA-1-B-B
7	MIB_3416	IR 93326:25-B-6-13-22-1RGA-2RGA-1-B-B
8	MIB_3417	IR 93327:1-B-7-21-25-1RGA-2RGA-1-B-B
9	MIB_3419	IR 93327:3-B-13-7-5-1RGA-2RGA-1-B-B
10	MIB_3421	IR 93327:5-B-15-3-8-1RGA-2RGA-1-B-B
11	MIB_3422	IR 93327:6-B-17-5-20-1RGA-2RGA-1-B-B
12	MIB_3423	IR 93327:7-B-16-4-9-1RGA-2RGA-1-B-B
13	MIB_3427	IR 93327:11-B-8-8-17-1RGA-2RGA-1-B-B
14	MIB_3432	IR 93327:17-B-5-13-12-1RGA-2RGA-1-B-B
15	MIB_3434	IR 93327:19-B-21-15-23-1RGA-2RGA-1-B-B
16	MIB_3438	IR 93327:24-B-7-12-12-1RGA-2RGA-1-B-B
17	MIB_3439	IR 93327:25-B-22-16-17-1RGA-2RGA-1-B-B
18	MIB_3441	IR 93327:27-B-8-19-6-1RGA-2RGA-1-B-B
19	MIB_3442	IR 93327:28-B-12-8-22-1RGA-2RGA-1-B-B
20	MIB_3446	IR 93327:32-B-19-25-12-1RGA-2RGA-1-B-B
21	MIB_3448	IR 93327:34-B-12-5-22-1RGA-2RGA-1-B-B
22	MIB_3450	IR 93327:36-B-23-8-15-1RGA-2RGA-1-B-B
23	MIB_3453	IR 93327:39-B-19-9-12-1RGA-2RGA-1-B-B
24	MIB_3456	IR 93327:42-B-17-17-13-1RGA-2RGA-1-B-B
25	MIB_3457	IR 93327:43-B-16-24-8-1RGA-2RGA-1-B-B
26	MIB_3466	IR 93328:10-B-8-12-19-1RGA-2RGA-1-B-B
27	MIB_3467	IR 93328:11-B-12-17-25-1RGA-2RGA-1-B-B
28	MIB_3469	IR 93328:13-B-8-6-23-1RGA-2RGA-1-B-B
29	MIB_3471	IR 93328:15-B-8-12-14-1RGA-2RGA-1-B-B
30	MIB_3474	IR 93328:18-B-14-12-22-1RGA-2RGA-1-B-B
31	MIB_3475	IR 93328:19-B-11-12-6-1RGA-2RGA-1-B-B
32	MIB_3476	IR 93328:20-B-8-22-7-1RGA-2RGA-1-B-B
33	MIB_3477	IR 93328:21-B-13-15-18-1RGA-2RGA-1-B-B
34	MIB_3478	IR 93328:22-B-14-15-6-1RGA-2RGA-1-B-B
35	MIB_3479	IR 93328:23-B-23-11-17-1RGA-2RGA-1-B-B
36	MIB_3480	IR 93328:24-B-25-8-15-1RGA-2RGA-1-B-B
37	MIB_3481	IR 93328:25-B-19-12-15-1RGA-2RGA-1-B-B
38	MIB_3483	IR 93328:27-B-19-8-21-1RGA-2RGA-1-B-B
39	MIB_3484	IR 93328:28-B-23-13-21-1RGA-2RGA-1-B-B

40	MIB_3487	IR 93328:31-B-13-3-13-1RGA-2RGA-1-B-B
41	MIB_3488	IR 93328:32-B-21-8-5-1RGA-2RGA-1-B-B
42	MIB_3494	IR 93328:38-B-17-14-11-1RGA-2RGA-1-B-B
43	MIB_3495	IR 93328:39-B-15-23-14-1RGA-2RGA-1-B-B
44	MIB_3496	IR 93328:40-B-15-25-16-1RGA-2RGA-1-B-B
45	MIB_3503	IR 93329:7-B-11-6-14-1RGA-2RGA-1-B-B
46	MIB_3504	IR 93329:8-B-14-17-16-1RGA-2RGA-1-B-B
47	MIB_3505	IR 93329:9-B-16-15-14-1RGA-2RGA-1-B-B
48	MIB_3506	IR 93329:10-B-15-15-2-1RGA-2RGA-1-B-B
49	MIB_3507	IR 93329:11-B-18-22-16-1RGA-2RGA-1-B-B
50	MIB_3508	IR 93329:12-B-14-21-11-1RGA-2RGA-1-B-B
51	MIB_3525	IR 93329:29-B-1-23-7-1RGA-2RGA-1-B-B
52	MIB_3532	IR 93329:36-B-21-9-14-1RGA-2RGA-1-B-B
53	MIB_3535	IR 93329:39-B-12-16-8-1RGA-2RGA-1-B-B
54	MIB_3538	IR 93329:42-B-14-16-18-1RGA-2RGA-1-B-B
55	MIB_3539	IR 93329:44-B-8-20-4-1RGA-2RGA-1-B-B
56	MIB_3540	IR 93329:45-B-14-13-10-1RGA-2RGA-1-B-B
57	MIB_3546	IR 93329:51-B-18-1-21-1RGA-2RGA-1-B-B
58	MIB_3548	IR 93329:53-B-4-21-23-1RGA-2RGA-1-B-B
59	MIB_3549	IR 93329:54-B-10-20-23-1RGA-2RGA-1-B-B
60	MIB_3553	IR 93329:58-B-12-21-4-1RGA-2RGA-1-B-B
61	MIB_3554	IR 93329:59-B-14-25-20-1RGA-2RGA-1-B-B
62	MIB_3556	IR 93329:61-B-21-12-21-1RGA-2RGA-1-B-B
63	MIB_3572	IR 93330:14-B-15-11-21-1RGA-2RGA-1-B-B
64	MIB_3573	IR 93330:15-B-16-11-18-1RGA-2RGA-1-B-B
65	MIB_3594	IR 93330:36-B-25-23-15-1RGA-2RGA-1-B-B
66	MIB_3595	IR 93330:37-B-9-15-16-1RGA-2RGA-1-B-B
67	MIB_3598	IR 93330:41-B-10-17-14-1RGA-2RGA-1-B-B
68	MIB_3603	IR 93330:46-B-16-18-17-1RGA-2RGA-1-B-B
69	MIB_3604	IR 93330:47-B-13-9-5-1RGA-2RGA-1-B-B
70	MIB_3606	IR 93330:49-B-14-19-16-1RGA-2RGA-1-B-B
71	MIB_3620	IR 93331:8-B-7-18-21-1RGA-2RGA-1-B-B
72	MIB_3621	IR 93331:9-B-10-10-12-1RGA-2RGA-1-B-B
73	MIB_3626	IR 93331:14-B-9-16-8-1RGA-2RGA-1-B-B
74	MIB_3629	IR 93331:17-B-12-14-20-1RGA-2RGA-1-B-B
75	MIB_3630	IR 93331:18-B-13-21-10-1RGA-2RGA-1-B-B
76	MIB_3636	IR 93331:24-B-17-16-18-1RGA-2RGA-1-B-B
77	MIB_3637	IR 93331:25-B-20-16-19-1RGA-2RGA-1-B-B
78	MIB_3644	IR 93331:32-B-18-10-8-1RGA-2RGA-1-B-B
79	MIB_3645	IR 93331:33-B-19-20-17-1RGA-2RGA-1-B-B
80	MIB_3668	IR 93332:22-B-22-4-18-1RGA-2RGA-1-B-B

81	MIB_3669	IR 93332:23-B-9-8-18-1RGA-2RGA-1-B-B
82	MIB_3672	IR 93332:26-B-22-17-18-1RGA-2RGA-1-B-B
83	MIB_3675	IR 93332:29-B-18-10-23-1RGA-2RGA-1-B-B
84	MIB_3677	IR 93332:31-B-14-5-19-1RGA-2RGA-1-B-B
85	MIB_3686	IR 93332:40-B-8-15-9-1RGA-2RGA-1-B-B
86	MIB_3688	IR 93332:42-B-12-19-6-1RGA-2RGA-1-B-B
87	MIB_3691	IR 93332:45-B-8-23-19-1RGA-2RGA-1-B-B
88	MIB_3693	IR 93333:1-B-10-3-20-1RGA-2RGA-1-B-B
89	MIB_3695	IR 93333:4-B-8-19-8-1RGA-2RGA-1-B-B
90	MIB_3707	IR 93333:18-B-19-7-16-1RGA-2RGA-1-B-B
91	MIB_3708	IR 93333:19-B-17-12-18-1RGA-2RGA-1-B-B
92	MIB_3726	IR 93333:40-B-12-12-22-1RGA-2RGA-1-B-B
93	MIB_3728	IR 93333:42-B-17-20-20-1RGA-2RGA-1-B-B
94	MIB_3732	IR 93334:1-B-20-12-9-1RGA-2RGA-1-B-B
95	MIB_3737	IR 93334:6-B-9-19-22-1RGA-2RGA-1-B-B
96	MIB_3739	IR 93334:8-B-10-8-4-1RGA-2RGA-1-B-B
97	MIB_3742	IR 93334:11-B-4-8-1-1RGA-2RGA-1-B-B
98	MIB_3743	IR 93334:12-B-20-12-1-1RGA-2RGA-1-B-B
99	MIB_3744	IR 93334:13-B-9-12-15-1RGA-2RGA-1-B-B
100	MIB_3769	IR 93334:45-B-22-7-15-1RGA-2RGA-1-B-B
101	MIB_3771	IR 93334:47-B-23-16-12-1RGA-2RGA-1-B-B
102	MIB_3774	IR 93334:50-B-12-21-5-1RGA-2RGA-1-B-B
103	MIB_3776	IR 93334:52-B-22-15-16-1RGA-2RGA-1-B-B
104	MIB_3785	IR 93334:61-B-13-16-19-1RGA-2RGA-1-B-B
105	MIB_3789	IR 93334:65-B-22-18-10-1RGA-2RGA-1-B-B
106	MIB_3795	IR 93335:4-B-3-6-7-1RGA-2RGA-1-B-B
107	MIB_3800	IR 93335:10-B-12-16-9-1RGA-2RGA-1-B-B
108	MIB_3801	IR 93335:11-B-5-21-11-1RGA-2RGA-1-B-B
109	MIB_3810	IR 93335:21-B-20-23-8-1RGA-2RGA-1-B-B
110	MIB_3811	IR 93335:22-B-12-13-13-1RGA-2RGA-1-B-B
111	MIB_3815	IR 93335:26-B-9-20-3-1RGA-2RGA-1-B-B
112	MIB_3819	IR 93335:30-B-13-3-11-1RGA-2RGA-1-B-B
113	MIB_3821	IR 93335:32-B-16-20-13-1RGA-2RGA-1-B-B
114	MIB_3823	IR 93335:34-B-13-8-16-1RGA-2RGA-1-B-B
115	MIB_3826	IR 93335:37-B-3-3-13-1RGA-2RGA-1-B-B
116	MIB_3828	IR 93335:39-B-4-8-3-1RGA-2RGA-1-B-B
117	MIB_3830	IR 93335:41-B-10-9-4-1RGA-2RGA-1-B-B
118	MIB_3831	IR 93335:42-B-21-11-8-1RGA-2RGA-1-B-B
119	MIB_3836	IR 93335:48-B-15-5-15-1RGA-2RGA-1-B-B
120	MIB_3838	IR 93335:51-B-9-8-10-1RGA-2RGA-1-B-B
121	MIB_3840	IR 93335:54-B-4-20-8-1RGA-2RGA-1-B-B

122	MIB_3846	IR 93335:60-B-17-12-19-1RGA-2RGA-1-B-B
123	MIB_3850	IR 93335:64-B-14-14-9-1RGA-2RGA-1-B-B
124	MIB_3852	IR 93335:66-B-17-17-14-1RGA-2RGA-1-B-B
125	MIB_3853	IR 93336:1-B-13-3-24-1RGA-2RGA-1-B-B
126	MIB_3865	IR 93336:15-B-10-9-18-1RGA-2RGA-1-B-B
127	MIB_3868	IR 93336:18-B-6-4-14-1RGA-2RGA-1-B-B
128	MIB_3872	IR 93336:23-B-15-21-6-1RGA-2RGA-1-B-B
129	MIB_3873	IR 93336:24-B-15-17-9-1RGA-2RGA-1-B-B
130	MIB_3877	IR 93336:28-B-14-14-15-1RGA-2RGA-1-B-B
131	MIB_3880	IR 93336:31-B-16-23-6-1RGA-2RGA-1-B-B
132	MIB_3897	IR 93336:50-B-5-13-20-1RGA-2RGA-1-B-B
133	MIB_3898	IR 93336:51-B-20-10-6-1RGA-2RGA-1-B-B
134	MIB_3901	IR 93336:54-B-3-6-10-1RGA-2RGA-1-B-B
135	MIB_3908	IR 93336:61-B-3-19-7-1RGA-2RGA-1-B-B
136	MIB_3910	IR 93336:63-B-20-8-2-1RGA-2RGA-1-B-B
137	MIB_3912	IR 93336:65-B-3-14-10-1RGA-2RGA-1-B-B
138	MIB_3917	IR 93336:70-B-19-11-3-1RGA-2RGA-1-B-B
139	MIB_3918	IR 93337:1-B-18-8-20-1RGA-2RGA-1-B-B
140	MIB_3920	IR 93337:4-B-19-10-19-1RGA-2RGA-1-B-B
141	MIB_3922	IR 93337:8-B-12-15-19-1RGA-2RGA-1-B-B
142	MIB_3924	IR 93337:10-B-20-6-19-1RGA-2RGA-1-B-B
143	MIB_3927	IR 93337:13-B-19-10-16-1RGA-2RGA-1-B-B
144	MIB_3930	IR 93337:16-B-11-22-20-1RGA-2RGA-1-B-B
145	MIB_3935	IR 93337:22-B-20-2-11-1RGA-2RGA-1-B-B
146	MIB_3939	IR 93337:26-B-12-24-14-1RGA-2RGA-1-B-B
147	MIB_3941	IR 93337:28-B-9-3-20-1RGA-2RGA-1-B-B
148	MIB_3942	IR 93337:29-B-20-3-21-1RGA-2RGA-1-B-B
149	MIB_3944	IR 93337:35-B-5-19-20-1RGA-2RGA-1-B-B
150	MIB_3945	IR 93337:36-B-14-20-13-1RGA-2RGA-1-B-B
151	MIB_3948	IR 93337:39-B-10-19-17-1RGA-2RGA-1-B-B
152	MIB_3955	IR 93337:48-B-18-20-13-1RGA-2RGA-1-B-B
153	MIB_3960	IR 93337:53-B-21-11-15-1RGA-2RGA-1-B-B
154	MIB_3961	IR 93337:54-B-18-18-18-1RGA-2RGA-1-B-B
155	MIB_3963	IR 93337:56-B-20-20-20-1RGA-2RGA-1-B-B
156	MIB_3964	IR 93337:57-B-8-14-5-1RGA-2RGA-1-B-B
157	MIB_3966	IR 93337:59-B-21-20-21-1RGA-2RGA-1-B-B
158	MIB_3967	IR 93337:60-B-14-21-18-1RGA-2RGA-1-B-B
159	MIB_3969	IR 93337:62-B-12-22-20-1RGA-2RGA-1-B-B
160	MIB_3980	IR 93338:10-B-15-10-20-1RGA-2RGA-1-B-B
161	MIB_3984	IR 93338:14-B-21-9-5-1RGA-2RGA-1-B-B
162	MIB_3986	IR 93338:16-B-14-15-15-1RGA-2RGA-1-B-B

163	MIB_3997	IR 93338:31-B-9-14-18-1RGA-2RGA-1-B-B
164	MIB_3999	IR 93338:33-B-21-12-14-1RGA-2RGA-1-B-B
165	MIB_4002	IR 93338:36-B-16-15-13-1RGA-2RGA-1-B-B
166	MIB_4015	IR 93338:50-B-18-10-6-1RGA-2RGA-1-B-B
167	MIB_4040	IR 93339:13-B-23-6-22-1RGA-2RGA-1-B-B
168	MIB_4046	IR 93339:23-B-18-17-18-1RGA-2RGA-1-B-B
169	MIB_4049	IR 93339:26-B-6-16-11-1RGA-2RGA-1-B-B
170	MIB_4050	IR 93339:27-B-11-21-13-1RGA-2RGA-1-B-B
171	MIB_4051	IR 93339:28-B-2-18-3-1RGA-2RGA-1-B-B
172	MIB_4053	IR 93339:31-B-17-4-19-1RGA-2RGA-1-B-B
173	MIB_4054	IR 93339:32-B-23-12-18-1RGA-2RGA-1-B-B
174	MIB_4062	IR 93339:42-B-20-19-10-1RGA-2RGA-1-B-B
175	MIB_4064	IR 93339:44-B-8-22-3-1RGA-2RGA-1-B-B
176	MIB_4070	IR 93339:50-B-20-22-10-1RGA-2RGA-1-B-B
177	MIB_4071	IR 93339:52-B-16-24-9-1RGA-2RGA-1-B-B
178	MIB_4072	IR 93339:53-B-8-23-8-1RGA-2RGA-1-B-B
179	MIB_4074	IR 93339:55-B-21-2-15-1RGA-2RGA-1-B-B
180	MIB_4075	IR 93339:56-B-16-5-13-1RGA-2RGA-1-B-B
181	MIB_4077	IR 93340:1-B-10-13-10-1RGA-2RGA-1-B-B
182	MIB_4082	IR 93340:6-B-20-18-12-1RGA-2RGA-1-B-B
183	MIB_4083	IR 93340:7-B-22-14-21-1RGA-2RGA-1-B-B
184	MIB_4086	IR 93340:10-B-11-11-10-1RGA-2RGA-1-B-B
185	MIB_4095	IR 93340:19-B-13-16-11-1RGA-2RGA-1-B-B
186	MIB_4126	IR 93340:60-B-4-5-19-1RGA-2RGA-1-B-B
187	MIB_4127	IR 93341:1-B-20-10-23-1RGA-2RGA-1-B-B
188	MIB_4131	IR 93341:9-B-19-10-4-1RGA-2RGA-1-B-B
189	MIB_4134	IR 93341:12-B-6-7-20-1RGA-2RGA-1-B-B
190	MIB_4135	IR 93341:13-B-2-21-21-1RGA-2RGA-1-B-B
191	MIB_4136	IR 93341:14-B-6-6-2-1RGA-2RGA-1-B-B
192	MIB_4137	IR 93341:16-B-10-11-3-1RGA-2RGA-1-B-B
193	MIB_4142	IR 93341:25-B-11-20-6-1RGA-2RGA-1-B-B
194	MIB_4153	IR 93341:37-B-20-22-3-1RGA-2RGA-1-B-B
195	MIB_4154	IR 93341:39-B-20-16-20-1RGA-2RGA-1-B-B
196	MIB_4159	IR 93341:44-B-12-21-13-1RGA-2RGA-1-B-B
197	MIB_4160	IR 93341:45-B-20-2-18-1RGA-2RGA-1-B-B
198	MIB_4161	IR 93341:46-B-18-21-18-1RGA-2RGA-1-B-B
199	MIB_4162	IR 93341:48-B-10-12-20-1RGA-2RGA-1-B-B
200	MIB_4163	IR 93341:49-B-9-13-9-1RGA-2RGA-1-B-B
201	MIB_4164	IR 93341:50-B-14-10-20-1RGA-2RGA-1-B-B
202	MIB_4167	IR 93341:53-B-11-19-21-1RGA-2RGA-1-B-B
203	MIB_4169	IR 93341:55-B-18-2-12-1RGA-2RGA-1-B-B

204	MIB_4172	IR 93341:59-B-3-13-10-1RGA-2RGA-1-B-B
205	MIB_4173	IR 93341:60-B-8-9-9-1RGA-2RGA-1-B-B
206	MIB_4176	IR 93342:3-B-4-5-20-1RGA-2RGA-1-B-B
207	MIB_4179	IR 93342:6-B-6-3-18-1RGA-2RGA-1-B-B
208	MIB_4180	IR 93342:7-B-7-10-23-1RGA-2RGA-1-B-B
209	MIB_4184	IR 93342:11-B-11-7-8-1RGA-2RGA-1-B-B
210	MIB_4188	IR 93342:15-B-23-18-22-1RGA-2RGA-1-B-B
211	MIB_4190	IR 93342:17-B-7-20-8-1RGA-2RGA-1-B-B
212	MIB_4193	IR 93342:20-B-7-20-7-1RGA-2RGA-1-B-B
213	MIB_4195	IR 93342:22-B-23-21-17-1RGA-2RGA-1-B-B
214	MIB_4198	IR 93342:25-B-10-20-6-1RGA-2RGA-1-B-B
215	MIB_4199	IR 93342:26-B-8-18-20-1RGA-2RGA-1-B-B
216	MIB_4203	IR 93342:32-B-18-14-9-1RGA-2RGA-1-B-B
217	MIB_4204	IR 93342:33-B-19-22-7-1RGA-2RGA-1-B-B
218	MIB_4208	IR 93342:37-B-21-18-8-1RGA-2RGA-1-B-B
219	MIB_4209	IR 93342:38-B-18-23-10-1RGA-2RGA-1-B-B
220	MIB_4220	IR 93343:11-B-8-7-20-1RGA-2RGA-1-B-B
221	MIB_4223	IR 93343:14-B-12-5-20-1RGA-2RGA-1-B-B
222	MIB_4224	IR 93343:15-B-9-11-16-1RGA-2RGA-1-B-B
223	MIB_4228	IR 93343:19-B-12-23-19-1RGA-2RGA-1-B-B
224	MIB_4233	IR 93343:24-B-14-7-16-1RGA-2RGA-1-B-B
225	MIB_4234	IR 93343:25-B-18-5-8-1RGA-2RGA-1-B-B
226	MIB_4235	IR 93343:26-B-5-23-8-1RGA-2RGA-1-B-B
227	MIB_4236	IR 93343:27-B-14-19-12-1RGA-2RGA-1-B-B
228	MIB_4241	IR 93343:32-B-13-8-17-1RGA-2RGA-1-B-B
229	MIB_4251	IR 93343:42-B-13-20-14-1RGA-2RGA-1-B-B
230	MIB_4252	IR 93343:43-B-16-21-17-1RGA-2RGA-1-B-B
231	MIB_4261	IR 93343:52-B-16-20-23-1RGA-2RGA-1-B-B
232	MIB_4265	IR 93343:56-B-17-8-17-1RGA-2RGA-1-B-B
233	MIB_4267	IR 93343:58-B-11-12-16-1RGA-2RGA-1-B-B
234	MIB_4270	IR 93344:3-B-6-13-17-1RGA-2RGA-1-B-B
235	MIB_4274	IR 93344:7-B-8-19-16-1RGA-2RGA-1-B-B
236	MIB_4279	IR 93344:12-B-3-14-6-1RGA-2RGA-1-B-B
237	MIB_4280	IR 93344:13-B-4-17-11-1RGA-2RGA-1-B-B
238	MIB_4286	IR 93344:21-B-3-23-7-1RGA-2RGA-1-B-B
239	MIB_4287	IR 93344:22-B-3-23-8-1RGA-2RGA-1-B-B
240	MIB_4288	IR 93344:23-B-16-22-8-1RGA-2RGA-1-B-B
241	MIB_4289	IR 93344:24-B-1-24-1-1RGA-2RGA-1-B-B
242	MIB_4290	IR 93344:25-B-20-10-20-1RGA-2RGA-1-B-B
243	MIB_4295	IR 93345:1-B-16-3-12-1RGA-2RGA-1-B-B
244	MIB_4299	IR 93345:6-B-21-21-6-1RGA-2RGA-1-B-B

245	MIB_4302	IR 93345:10-B-4-23-16-1RGA-2RGA-1-B-B
246	MIB_4305	IR 93345:13-B-8-17-11-1RGA-2RGA-1-B-B
247	MIB_4309	IR 93345:17-B-6-22-16-1RGA-2RGA-1-B-B
248	MIB_4316	IR 93345:24-B-10-8-3-1RGA-2RGA-1-B-B
249	MIB_4320	IR 93345:28-B-9-18-6-1RGA-2RGA-1-B-B
250	MIB_4322	IR 93345:31-B-2-4-6-1RGA-2RGA-1-B-B
251	MIB_4325	IR 93346:2-B-5-9-8-1RGA-2RGA-1-B-B
252	MIB_4328	IR 93346:5-B-3-3-7-1RGA-2RGA-1-B-B
253	MIB_4332	IR 93346:10-B-2-11-14-1RGA-2RGA-1-B-B
254	MIB_4338	IR 93346:16-B-15-7-10-1RGA-2RGA-1-B-B
255	MIB_4339	IR 93346:17-B-7-2-6-1RGA-2RGA-1-B-B
256	MIB_4340	IR 93346:18-B-24-12-5-1RGA-2RGA-1-B-B
257	MIB_4341	IR 93346:19-B-7-21-13-1RGA-2RGA-1-B-B
258	MIB_4345	IR 93346:23-B-10-4-3-1RGA-2RGA-1-B-B
259	MIB_4347	IR 93346:25-B-7-6-18-1RGA-2RGA-1-B-B
260	MIB_4348	IR 93346:26-B-20-8-2-1RGA-2RGA-1-B-B
261	MIB_4350	IR 93346:28-B-19-6-2-1RGA-2RGA-1-B-B
262	MIB_4352	IR 93346:31-B-9-11-8-1RGA-2RGA-1-B-B
263	MIB_4353	IR 93346:33-B-10-8-18-1RGA-2RGA-1-B-B
264	MIB_4354	IR 93346:35-B-23-2-7-1RGA-2RGA-1-B-B
265	MIB_4356	IR 93347:1-B-8-4-7-1RGA-2RGA-1-B-B
266	MIB_4357	IR 93347:2-B-10-14-4-1RGA-2RGA-1-B-B
267	MIB_4358	IR 93347:3-B-11-4-10-1RGA-2RGA-1-B-B
268	MIB_4359	IR 93347:4-B-9-9-22-1RGA-2RGA-1-B-B
269	MIB_4360	IR 93347:5-B-7-3-10-1RGA-2RGA-1-B-B
270	MIB_4361	IR 93347:6-B-21-5-6-1RGA-2RGA-1-B-B
271	MIB_4363	IR 93347:8-B-12-10-20-1RGA-2RGA-1-B-B
272	MIB_4365	IR 93347:10-B-23-5-19-1RGA-2RGA-1-B-B
273	MIB_4367	IR 93347:12-B-3-5-8-1RGA-2RGA-1-B-B
274	MIB_4369	IR 93347:14-B-8-3-22-1RGA-2RGA-1-B-B
275	MIB_4370	IR 93347:15-B-14-3-10-1RGA-2RGA-1-B-B
276	MIB_4372	IR 93347:17-B-4-18-23-1RGA-2RGA-1-B-B
277	MIB_4374	IR 93347:19-B-3-2-21-1RGA-2RGA-1-B-B
278	MIB_4376	IR 93347:21-B-7-7-5-1RGA-2RGA-1-B-B
279	MIB_4379	IR 93348:1-B-21-18-9-1RGA-2RGA-1-B-B
280	MIB_4383	IR 93348:5-B-11-8-12-1RGA-2RGA-1-B-B
281	MIB_4388	IR 93348:10-B-5-10-11-1RGA-2RGA-1-B-B
282	MIB_4389	IR 93348:11-B-5-22-8-1RGA-2RGA-1-B-B
283	MIB_4393	IR 93348:15-B-16-20-21-1RGA-2RGA-1-B-B
284	MIB_4395	IR 93348:17-B-19-19-8-1RGA-2RGA-1-B-B
285	MIB_4410	IR 93348:32-B-15-9-5-1RGA-2RGA-1-B-B

286	MIB_4411	IR 93348:34-B-22-21-14-1RGA-2RGA-1-B-B
287	MIB_4414	IR 93348:37-B-19-2-19-1RGA-2RGA-1-B-B
288	MIB_4417	IR 93348:43-B-1-14-13-1RGA-2RGA-1-B-B
289	MIB_4418	IR 93348:44-B-6-4-22-1RGA-2RGA-1-B-B
290	MIB_4424	IR 93349:4-B-11-21-22-1RGA-2RGA-1-B-B
291	MIB_4432	IR 93349:12-B-5-5-19-1RGA-2RGA-1-B-B
292	MIB_4433	IR 93349:13-B-8-5-4-1RGA-2RGA-1-B-B
293	MIB_4434	IR 93349:14-B-6-5-4-1RGA-2RGA-1-B-B
294	MIB_4438	IR 93349:18-B-21-16-1-1RGA-2RGA-1-B-B
295	MIB_4439	IR 93349:19-B-8-19-6-1RGA-2RGA-1-B-B
296	MIB_4445	IR 93349:27-B-23-6-20-1RGA-2RGA-1-B-B
297	MIB_4447	IR 93349:30-B-6-20-10-1RGA-2RGA-1-B-B
298	MIB_4448	IR 93349:31-B-4-11-21-1RGA-2RGA-1-B-B
299	MIB_4449	IR 93349:32-B-9-4-8-1RGA-2RGA-1-B-B
300	MIB_4450	IR 93349:33-B-19-12-7-1RGA-2RGA-1-B-B
301	MIB_4454	IR 93349:37-B-23-19-8-1RGA-2RGA-1-B-B
302	MIB_4455	IR 93349:38-B-16-15-6-1RGA-2RGA-1-B-B
303	MIB_4456	IR 93349:39-B-8-7-22-1RGA-2RGA-1-B-B
304	MIB_4462	IR 93349:46-B-16-2-4-1RGA-2RGA-1-B-B
305	MIB_4465	IR 93349:49-B-7-1-2-1RGA-2RGA-1-B-B
306	MIB_4467	IR 93349:51-B-18-12-23-1RGA-2RGA-1-B-B
307	MIB_4472	IR 93350:1-B-17-5-19-1RGA-2RGA-1-B-B
308	MIB_4473	IR 93350:2-B-21-20-23-1RGA-2RGA-1-B-B
309	MIB_4474	IR 93350:3-B-4-11-21-1RGA-2RGA-1-B-B
310	MIB_4479	IR 93350:8-B-8-7-23-1RGA-2RGA-1-B-B
311	MIB_4481	IR 93350:10-B-2-7-9-1RGA-2RGA-1-B-B
312	MIB_4482	IR 93350:11-B-2-5-18-1RGA-2RGA-1-B-B
313	MIB_4485	IR 93350:14-B-8-22-16-1RGA-2RGA-1-B-B
314	MIB_4488	IR 93350:17-B-9-21-7-1RGA-2RGA-1-B-B
315	MIB_4489	IR 93350:18-B-13-8-12-1RGA-2RGA-1-B-B
316	MIB_4493	IR 93350:22-B-18-22-12-1RGA-2RGA-1-B-B
317	MIB_4497	IR 93350:26-B-12-23-21-1RGA-2RGA-1-B-B
318	MIB_4502	IR 93350:31-B-21-9-21-1RGA-2RGA-1-B-B
319	MIB_4506	IR 93350:35-B-14-19-2-1RGA-2RGA-1-B-B
320	MIB_4507	IR 93350:36-B-10-23-9-1RGA-2RGA-1-B-B
321	MIB_4508	IR 93350:37-B-10-16-4-1RGA-2RGA-1-B-B
322	MIB_4514	IR 93350:43-B-23-16-12-1RGA-2RGA-1-B-B
323	MIB_4516	IR 93350:45-B-23-16-15-1RGA-2RGA-1-B-B
324	MIB_4518	IR 93350:47-B-10-23-7-1RGA-2RGA-1-B-B
325	MIB_4520	IR 93350:49-B-24-12-14-1RGA-2RGA-1-B-B
326	MIB_4524	IR 93351:1-B-2-12-8-1RGA-2RGA-1-B-B

327	MIB_4529	IR 93351:6-B-7-4-7-1RGA-2RGA-1-B-B
328	MIB_4532	IR 93351:9-B-6-5-10-1RGA-2RGA-1-B-B
329	MIB_4538	IR 93352:5-B-20-9-23-1RGA-2RGA-1-B-B
330	MIB_4543	IR 93353:5-B-9-9-23-1RGA-2RGA-1-B-B
331	MIB_4545	IR 93353:7-B-1-12-19-1RGA-2RGA-1-B-B
332	MIB_4546	IR 93353:8-B-1-5-9-1RGA-2RGA-1-B-B
333	MIB_4551	IR 93353:13-B-17-14-24-1RGA-2RGA-1-B-B
334	MIB_4552	IR 93353:14-B-8-12-8-1RGA-2RGA-1-B-B
335	MIB_4553	IR 93353:15-B-3-22-7-1RGA-2RGA-1-B-B
336	MIB_4555	IR 93353:17-B-18-8-12-1RGA-2RGA-1-B-B
337	MIB_4556	IR 93353:18-B-16-21-6-1RGA-2RGA-1-B-B
338	MIB_4557	IR 93353:19-B-21-21-2-1RGA-2RGA-1-B-B
339	MIB_4562	IR 93353:24-B-23-10-20-1RGA-2RGA-1-B-B
340	MIB_4566	IR 93353:28-B-23-23-21-1RGA-2RGA-1-B-B
341	MIB_4570	IR 93353:32-B-21-17-1-1RGA-2RGA-1-B-B
342	MIB_4572	IR 93353:34-B-17-7-19-1RGA-2RGA-1-B-B
343	MIB_4576	IR 93353:38-B-6-19-8-1RGA-2RGA-1-B-B
344	MIB_4579	IR 93353:41-B-20-2-18-1RGA-2RGA-1-B-B
345	MIB_4585	IR 93353:48-B-21-12-23-1RGA-2RGA-1-B-B
346	MIB_4588	IR 93353:51-B-8-4-9-1RGA-2RGA-1-B-B
347	MIB_4591	IR 93353:54-B-12-7-22-1RGA-2RGA-1-B-B
348	MIB_4596	IR 93354:5-B-4-9-17-1RGA-2RGA-1-B-B
349	MIB_4605	IR 93354:14-B-9-8-6-1RGA-2RGA-1-B-B
350	MIB_4606	IR 93354:15-B-3-3-24-1RGA-2RGA-1-B-B
351	MIB_4607	IR 93354:16-B-13-21-19-1RGA-2RGA-1-B-B
352	MIB_4608	IR 93354:17-B-5-18-17-1RGA-2RGA-1-B-B
353	MIB_4609	IR 93354:18-B-22-16-21-1RGA-2RGA-1-B-B
354	MIB_4611	IR 93354:20-B-17-3-8-1RGA-2RGA-1-B-B
355	MIB_4619	IR 93354:28-B-19-23-14-1RGA-2RGA-1-B-B
356	MIB_4620	IR 93354:29-B-5-7-9-1RGA-2RGA-1-B-B
357	MIB_4623	IR 93354:32-B-19-20-18-1RGA-2RGA-1-B-B
358	MIB_4627	IR 93355:2-B-12-5-13-1RGA-2RGA-1-B-B
359	MIB_4628	IR 93355:3-B-12-20-8-1RGA-2RGA-1-B-B
360	MIB_4632	IR 93356:4-B-20-11-13-1RGA-2RGA-1-B-B
361	MIB_4634	IR 93356:6-B-11-6-22-1RGA-2RGA-1-B-B
362	MIB_4635	IR 93356:7-B-5-24-12-1RGA-2RGA-1-B-B
363	MIB_4641	IR 93356:13-B-8-8-2-1RGA-2RGA-1-B-B
364	MIB_4642	IR 93356:14-B-4-8-8-1RGA-2RGA-1-B-B
365	MIB_4643	IR 93356:15-B-3-19-11-1RGA-2RGA-1-B-B
366	MIB_4648	IR 93356:20-B-3-14-19-1RGA-2RGA-1-B-B
367	MIB_4650	IR 93356:23-B-8-6-18-1RGA-2RGA-1-B-B

368	MIB_4651	IR 93356:25-B-5-5-12-1RGA-2RGA-1-B-B
369	MIB_4656	IR 93356:31-B-3-12-20-1RGA-2RGA-1-B-B
370	MIB_4660	IR 93356:35-B-22-13-7-1RGA-2RGA-1-B-B
371	MIB_4661	IR 93356:36-B-3-5-19-1RGA-2RGA-1-B-B
372	MIB_4665	IR 93357:4-B-8-3-8-1RGA-2RGA-1-B-B
373	MIB_4671	IR 93357:10-B-13-11-2-1RGA-2RGA-1-B-B
374	MIB_4672	IR 93357:11-B-7-19-3-1RGA-2RGA-1-B-B
375	MIB_4673	IR 93357:12-B-5-5-20-1RGA-2RGA-1-B-B
376	MIB_4674	IR 93357:13-B-7-9-4-1RGA-2RGA-1-B-B
377	MIB_4676	IR 93357:15-B-8-19-2-1RGA-2RGA-1-B-B
378	MIB_4679	IR 93357:18-B-8-18-10-1RGA-2RGA-1-B-B
379	MIB_4680	IR 93358:1-B-12-6-4-1RGA-2RGA-1-B-B
380	MIB_4682	IR 93358:3-B-3-12-11-1RGA-2RGA-1-B-B
381	MIB_4683	IR 93358:4-B-21-4-3-1RGA-2RGA-1-B-B
382	MIB_4691	IR 93358:12-B-8-7-23-1RGA-2RGA-1-B-B
383	MIB_4697	IR 93358:18-B-8-4-7-1RGA-2RGA-1-B-B
384	MIB_4699	IR 93358:20-B-5-20-7-1RGA-2RGA-1-B-B
385	MIB_4700	IR 93358:21-B-3-16-5-1RGA-2RGA-1-B-B
386	MIB_4701	IR 93358:22-B-21-16-7-1RGA-2RGA-1-B-B
387	MIB_4707	IR 93358:28-B-22-2-12-1RGA-2RGA-1-B-B
388	MIB_4712	IR 93342:39-B-20-1-11-1RGA-2RGA-1-B-B
389	MIB_4716	IR 93335:68-B-11-13-22-1RGA-2RGA-1-B-B
390	MIB_4718	IR 93336:72-B-15-4-8-1RGA-2RGA-1-B-B
391	MIB_4719	IR 93354:35-B-18-17-12-1RGA-2RGA-1-B-B

Supplementary Table S 2 : BPH resistance scores of 391 RiMi population across years

S.No.	Entry	2016	2017	Mean over years
1	MIB_3400	9.0	9.0	9.0
2	MIB_3406	6.9	7.1	7.0
3	MIB_3408	8.2	8.4	8.3
4	MIB_3409	2.8	2.2	2.5
5	MIB_3414	8.1	7.9	8.0
6	MIB_3415	8.4	8.3	8.3
7	MIB_3416	9.0	9.0	9.0
8	MIB_3417	8.6	8.6	8.6
9	MIB_3419	8.3	8.1	8.2
10	MIB_3421	7.1	6.9	7.0
11	MIB_3422	7.8	7.7	7.8
12	MIB_3423	7.6	7.5	7.5
13	MIB_3427	9.0	9.0	9.0
14	MIB_3432	8.4	8.2	8.3
15	MIB_3434	8.5	8.2	8.3
16	MIB_3438	9.0	9.0	9.0
17	MIB_3439	9.0	9.0	9.0
18	MIB_3441	8.4	8.3	8.4
19	MIB_3442	9.0	9.0	9.0
20	MIB_3446	9.0	9.0	9.0
21	MIB_3448	8.8	8.8	8.8
22	MIB_3450	9.0	9.0	9.0
23	MIB_3453	9.0	9.0	9.0
24	MIB_3456	9.0	9.0	9.0
25	MIB_3457	9.0	9.0	9.0
26	MIB_3466	9.0	9.0	9.0
27	MIB_3467	9.0	9.0	9.0
28	MIB_3469	9.0	9.0	9.0
29	MIB_3471	9.0	9.0	9.0
30	MIB_3474	9.0	9.0	9.0
31	MIB_3475	9.0	9.0	9.0
32	MIB_3476	9.0	9.0	9.0
33	MIB_3477	9.0	9.0	9.0
34	MIB_3478	9.0	9.0	9.0
35	MIB_3479	9.0	9.0	9.0
36	MIB_3480	9.0	9.0	9.0
37	MIB_3481	9.0	9.0	9.0
38	MIB_3483	9.0	9.0	9.0
39	MIB_3484	9.0	9.0	9.0

40	MIB_3487	9.0	9.0	9.0
41	MIB_3488	6.7	6.5	6.6
42	MIB_3494	7.4	7.2	7.3
43	MIB_3495	7.1	7.0	7.0
44	MIB_3496	7.1	6.9	7.0
45	MIB_3503	8.4	8.2	8.3
46	MIB_3504	8.6	8.6	8.6
47	MIB_3505	9.0	9.0	9.0
48	MIB_3506	8.5	8.5	8.5
49	MIB_3507	8.2	8.0	8.1
50	MIB_3508	9.0	9.0	9.0
51	MIB_3525	7.8	7.6	7.7
52	MIB_3532	8.4	8.3	8.3
53	MIB_3535	7.8	7.7	7.7
54	MIB_3538	7.3	7.2	7.2
55	MIB_3539	7.9	7.7	7.8
56	MIB_3540	7.5	7.3	7.4
57	MIB_3546	5.5	5.4	5.5
58	MIB_3548	7.5	7.4	7.4
59	MIB_3549	8.5	8.5	8.5
60	MIB_3553	5.5	5.3	5.4
61	MIB_3554	4.0	2.8	3.4
62	MIB_3556	6.0	6.4	6.2
63	MIB_3572	8.4	8.2	8.3
64	MIB_3573	8.0	7.9	7.9
65	MIB_3594	7.2	7.1	7.2
66	MIB_3595	7.2	7.0	7.1
67	MIB_3598	6.2	6.0	6.1
68	MIB_3603	7.1	7.0	7.1
69	MIB_3604	6.1	6.1	6.1
70	MIB_3606	5.7	5.7	5.7
71	MIB_3620	7.2	7.1	7.1
72	MIB_3621	7.1	6.9	7.0
73	MIB_3626	8.1	8.0	8.1
74	MIB_3629	9.0	9.0	9.0
75	MIB_3630	6.9	6.8	6.9
76	MIB_3636	9.0	9.0	9.0
77	MIB_3637	7.4	7.3	7.3
78	MIB_3644	7.1	7.0	7.0
79	MIB_3645	7.1	6.9	7.0
80	MIB_3668	3.4	3.0	3.2

81	MIB_3669	9.0	9.0	9.0
82	MIB_3672	9.0	9.0	9.0
83	MIB_3675	9.0	9.0	9.0
84	MIB_3677	9.0	9.0	9.0
85	MIB_3686	9.0	9.0	9.0
86	MIB_3688	9.0	9.0	9.0
87	MIB_3691	9.0	9.0	9.0
88	MIB_3693	1.6	1.6	1.6
89	MIB_3695	9.0	9.0	9.0
90	MIB_3707	6.4	5.2	5.8
91	MIB_3708	8.1	7.9	8.0
92	MIB_3726	7.3	7.1	7.2
93	MIB_3728	9.0	9.0	9.0
94	MIB_3732	9.0	9.0	9.0
95	MIB_3737	9.0	9.0	9.0
96	MIB_3739	9.0	9.0	9.0
97	MIB_3742	9.0	9.0	9.0
98	MIB_3743	9.0	9.0	9.0
99	MIB_3744	9.0	9.0	9.0
100	MIB_3769	9.0	9.0	9.0
101	MIB_3771	9.0	9.0	9.0
102	MIB_3774	9.0	9.0	9.0
103	MIB_3776	8.4	8.3	8.4
104	MIB_3785	8.2	8.1	8.2
105	MIB_3789	8.7	8.7	8.7
106	MIB_3795	8.5	8.5	8.5
107	MIB_3800	8.8	8.8	8.8
108	MIB_3801	9.0	9.0	9.0
109	MIB_3810	7.4	7.2	7.3
110	MIB_3811	8.8	8.8	8.8
111	MIB_3815	8.2	8.0	8.1
112	MIB_3819	7.3	7.2	7.3
113	MIB_3821	7.7	7.5	7.6
114	MIB_3823	8.2	8.1	8.1
115	MIB_3826	8.5	8.5	8.5
116	MIB_3828	9.0	9.0	9.0
117	MIB_3830	9.0	9.0	9.0
118	MIB_3831	9.0	9.0	9.0
119	MIB_3836	9.0	9.0	9.0
120	MIB_3838	9.0	9.0	9.0
121	MIB_3840	7.8	7.6	7.7

122	MIB_3846	7.5	7.3	7.4
123	MIB_3850	2.4	2.4	2.4
124	MIB_3852	8.6	8.6	8.6
125	MIB_3853	9.0	9.0	9.0
126	MIB_3865	8.4	8.2	8.3
127	MIB_3868	7.6	7.4	7.5
128	MIB_3872	7.3	7.1	7.2
129	MIB_3873	9.0	9.0	9.0
130	MIB_3877	9.0	9.0	9.0
131	MIB_3880	9.0	9.0	9.0
132	MIB_3897	9.0	9.0	9.0
133	MIB_3898	9.0	9.0	9.0
134	MIB_3901	9.0	9.0	9.0
135	MIB_3908	9.0	9.0	9.0
136	MIB_3910	9.0	9.0	9.0
137	MIB_3912	7.9	7.8	7.8
138	MIB_3917	9.0	9.0	9.0
139	MIB_3918	8.7	8.7	8.7
140	MIB_3920	9.0	9.0	9.0
141	MIB_3922	8.6	8.6	8.6
142	MIB_3924	9.0	9.0	9.0
143	MIB_3927	9.0	9.0	9.0
144	MIB_3930	9.0	9.0	9.0
145	MIB_3935	8.4	8.3	8.3
146	MIB_3939	7.5	7.4	7.5
147	MIB_3941	9.0	9.0	9.0
148	MIB_3942	8.3	8.2	8.3
149	MIB_3944	8.1	8.0	8.0
150	MIB_3945	9.0	9.0	9.0
151	MIB_3948	9.0	9.0	9.0
152	MIB_3955	8.1	7.9	8.0
153	MIB_3960	7.6	7.5	7.6
154	MIB_3961	8.2	8.1	8.2
155	MIB_3963	7.2	7.0	7.1
156	MIB_3964	7.4	7.3	7.4
157	MIB_3966	7.1	7.0	7.1
158	MIB_3967	8.0	7.8	7.9
159	MIB_3969	7.1	6.9	7.0
160	MIB_3980	8.1	8.0	8.1
161	MIB_3984	9.0	9.0	9.0
162	MIB_3986	8.0	7.9	8.0

163	MIB_3997	8.7	8.7	8.7
164	MIB_3999	7.2	7.1	7.2
165	MIB_4002	8.4	8.2	8.3
166	MIB_4015	8.1	7.9	8.0
167	MIB_4040	9.0	9.0	9.0
168	MIB_4046	8.7	8.7	8.7
169	MIB_4049	9.0	9.0	9.0
170	MIB_4050	9.0	9.0	9.0
171	MIB_4051	7.4	7.2	7.3
172	MIB_4053	8.2	8.0	8.1
173	MIB_4054	7.3	7.2	7.2
174	MIB_4062	5.6	5.4	5.5
175	MIB_4064	8.7	8.7	8.7
176	MIB_4070	7.8	7.7	7.8
177	MIB_4071	7.5	7.3	7.4
178	MIB_4072	3.6	3.6	3.6
179	MIB_4074	8.3	8.1	8.2
180	MIB_4075	6.9	6.7	6.8
181	MIB_4077	2.2	1.3	1.7
182	MIB_4082	6.2	6.1	6.2
183	MIB_4083	7.9	7.7	7.8
184	MIB_4086	6.3	6.2	6.3
185	MIB_4095	5.7	5.6	5.6
186	MIB_4126	4.4	3.8	4.1
187	MIB_4127	6.2	6.0	6.1
188	MIB_4131	4.0	3.5	3.7
189	MIB_4134	3.2	2.1	2.6
190	MIB_4135	5.5	5.3	5.4
191	MIB_4136	6.6	6.4	6.5
192	MIB_4137	6.2	6.1	6.1
193	MIB_4142	5.4	5.3	5.3
194	MIB_4153	7.2	7.0	7.1
195	MIB_4154	5.9	5.8	5.8
196	MIB_4159	8.5	8.5	8.5
197	MIB_4160	9.0	9.0	9.0
198	MIB_4161	8.2	8.1	8.1
199	MIB_4162	6.5	6.4	6.4
200	MIB_4163	4.1	4.1	4.1
201	MIB_4164	5.8	5.7	5.7
202	MIB_4167	5.6	5.5	5.6
203	MIB_4169	7.7	7.6	7.7

204	MIB_4172	9.0	9.0	9.0
205	MIB_4173	8.4	8.3	8.4
206	MIB_4176	6.1	5.9	6.0
207	MIB_4179	8.1	8.0	8.0
208	MIB_4180	8.0	7.8	7.9
209	MIB_4184	9.0	9.0	9.0
210	MIB_4188	7.9	7.8	7.9
211	MIB_4190	7.3	7.1	7.2
212	MIB_4193	8.1	7.9	8.0
213	MIB_4195	7.6	7.4	7.5
214	MIB_4198	7.5	7.4	7.4
215	MIB_4199	7.0	6.9	7.0
216	MIB_4203	6.6	6.5	6.6
217	MIB_4204	5.7	5.5	5.6
218	MIB_4208	2.7	2.0	2.4
219	MIB_4209	7.7	7.5	7.6
220	MIB_4220	7.6	7.5	7.5
221	MIB_4223	7.2	7.1	7.1
222	MIB_4224	7.7	7.6	7.6
223	MIB_4228	5.9	5.7	5.8
224	MIB_4233	7.5	7.3	7.4
225	MIB_4234	7.0	6.8	6.9
226	MIB_4235	4.4	6.5	5.4
227	MIB_4236	5.7	5.6	5.7
228	MIB_4241	2.5	1.9	2.2
229	MIB_4251	5.9	7.3	6.6
230	MIB_4252	7.3	7.2	7.3
231	MIB_4261	7.7	7.5	7.6
232	MIB_4265	8.5	8.5	8.5
233	MIB_4267	7.3	7.1	7.2
234	MIB_4270	6.7	6.6	6.7
235	MIB_4274	5.7	5.5	5.6
236	MIB_4279	7.8	7.6	7.7
237	MIB_4280	5.6	5.4	5.5
238	MIB_4286	3.2	2.2	2.7
239	MIB_4287	6.6	6.4	6.5
240	MIB_4288	8.2	8.0	8.1
241	MIB_4289	7.4	7.3	7.3
242	MIB_4290	5.7	5.6	5.6
243	MIB_4295	8.3	8.2	8.2
244	MIB_4299	9.0	9.0	9.0

245	MIB_4302	8.3	8.1	8.2
246	MIB_4305	8.5	8.5	8.5
247	MIB_4309	9.0	9.0	9.0
248	MIB_4316	8.7	8.7	8.7
249	MIB_4320	6.4	7.9	7.2
250	MIB_4322	9.0	9.0	9.0
251	MIB_4325	8.2	8.1	8.2
252	MIB_4328	9.0	9.0	9.0
253	MIB_4332	8.3	8.2	8.3
254	MIB_4338	9.0	9.0	9.0
255	MIB_4339	8.9	8.9	8.9
256	MIB_4340	8.3	8.1	8.2
257	MIB_4341	8.4	8.2	8.3
258	MIB_4345	9.0	9.0	9.0
259	MIB_4347	7.0	6.8	6.9
260	MIB_4348	8.6	8.6	8.6
261	MIB_4350	9.0	9.0	9.0
262	MIB_4352	8.4	8.8	8.6
263	MIB_4353	9.0	8.6	8.8
264	MIB_4354	8.1	8.0	8.1
265	MIB_4356	8.1	7.9	8.0
266	MIB_4357	7.6	7.4	7.5
267	MIB_4358	5.2	5.1	5.2
268	MIB_4359	7.5	7.4	7.5
269	MIB_4360	8.7	8.7	8.7
270	MIB_4361	6.2	6.0	6.1
271	MIB_4363	8.3	8.2	8.2
272	MIB_4365	8.7	8.7	8.7
273	MIB_4367	3.0	3.0	3.0
274	MIB_4369	8.7	8.7	8.7
275	MIB_4370	2.6	1.8	2.2
276	MIB_4372	9.0	9.0	9.0
277	MIB_4374	7.2	7.0	7.1
278	MIB_4376	6.2	6.1	6.2
279	MIB_4379	5.8	5.6	5.7
280	MIB_4383	5.9	5.8	5.9
281	MIB_4388	4.7	4.9	4.8
282	MIB_4389	5.7	5.7	5.7
283	MIB_4393	2.0	0.8	1.4
284	MIB_4395	5.4	5.2	5.3
285	MIB_4410	6.2	7.4	6.8

286	MIB_4411	5.9	7.8	6.8
287	MIB_4414	6.9	8.0	7.4
288	MIB_4417	6.3	6.1	6.2
289	MIB_4418	5.5	5.4	5.5
290	MIB_4424	4.2	3.8	4.0
291	MIB_4432	5.6	5.5	5.5
292	MIB_4433	7.7	7.6	7.7
293	MIB_4434	5.6	5.4	5.5
294	MIB_4438	7.1	7.0	7.0
295	MIB_4439	6.1	6.0	6.1
296	MIB_4445	5.6	5.5	5.6
297	MIB_4447	5.6	5.4	5.5
298	MIB_4448	8.6	8.6	8.6
299	MIB_4449	7.6	7.5	7.6
300	MIB_4450	5.8	5.7	5.8
301	MIB_4454	8.1	8.0	8.0
302	MIB_4455	7.9	7.7	7.8
303	MIB_4456	8.8	8.8	8.8
304	MIB_4462	8.4	8.2	8.3
305	MIB_4465	8.5	8.5	8.5
306	MIB_4467	8.4	8.3	8.4
307	MIB_4472	8.5	8.5	8.5
308	MIB_4473	6.8	6.7	6.8
309	MIB_4474	5.1	4.9	5.0
310	MIB_4479	8.0	7.9	7.9
311	MIB_4481	7.9	7.8	7.8
312	MIB_4482	6.4	6.4	6.4
313	MIB_4485	8.3	8.1	8.2
314	MIB_4488	6.2	6.0	6.1
315	MIB_4489	8.3	8.2	8.3
316	MIB_4493	7.5	7.3	7.4
317	MIB_4497	7.5	7.4	7.4
318	MIB_4502	8.6	8.6	8.6
319	MIB_4506	7.3	7.2	7.2
320	MIB_4507	7.9	7.7	7.8
321	MIB_4508	8.5	8.5	8.5
322	MIB_4514	7.5	7.3	7.4
323	MIB_4516	7.2	7.1	7.2
324	MIB_4518	9.0	9.0	9.0
325	MIB_4520	8.2	8.1	8.1
326	MIB_4524	7.9	7.8	7.9

327	MIB_4529	8.3	8.1	8.2
328	MIB_4532	8.5	8.5	8.5
329	MIB_4538	8.5	8.5	8.5
330	MIB_4543	8.4	8.2	8.3
331	MIB_4545	6.0	5.8	5.9
332	MIB_4546	7.9	7.7	7.8
333	MIB_4551	9.0	9.0	9.0
334	MIB_4552	8.1	7.9	8.0
335	MIB_4553	8.4	8.3	8.3
336	MIB_4555	7.2	7.0	7.1
337	MIB_4556	8.0	7.8	7.9
338	MIB_4557	5.4	5.3	5.4
339	MIB_4562	6.7	6.5	6.6
340	MIB_4566	6.3	6.2	6.2
341	MIB_4570	2.1	1.8	1.9
342	MIB_4572	8.8	8.8	8.8
343	MIB_4576	7.8	7.7	7.7
344	MIB_4579	6.8	6.6	6.7
345	MIB_4585	7.5	7.4	7.5
346	MIB_4588	7.1	6.9	7.0
347	MIB_4591	7.9	7.8	7.8
348	MIB_4596	7.9	7.7	7.8
349	MIB_4605	8.8	8.8	8.8
350	MIB_4606	6.2	6.1	6.1
351	MIB_4607	9.0	9.0	9.0
352	MIB_4608	7.2	7.1	7.1
353	MIB_4609	8.7	8.7	8.7
354	MIB_4611	8.5	8.5	8.5
355	MIB_4619	2.9	2.0	2.5
356	MIB_4620	7.4	7.2	7.3
357	MIB_4623	5.4	5.2	5.3
358	MIB_4627	4.1	4.1	4.1
359	MIB_4628	7.4	7.3	7.4
360	MIB_4632	5.9	5.7	5.8
361	MIB_4634	5.2	5.2	5.2
362	MIB_4635	8.0	7.9	8.0
363	MIB_4641	7.4	7.2	7.3
364	MIB_4642	5.5	5.7	5.6
365	MIB_4643	6.5	8.2	7.3
366	MIB_4648	6.7	6.6	6.6
367	MIB_4650	9.0	9.0	9.0

368	MIB_4651	9.0	9.0	9.0
369	MIB_4656	9.0	9.0	9.0
370	MIB_4660	8.7	8.7	8.7
371	MIB_4661	7.8	7.6	7.7
372	MIB_4665	7.3	7.1	7.2
373	MIB_4671	7.2	7.0	7.1
374	MIB_4672	5.6	5.5	5.5
375	MIB_4673	7.7	7.5	7.6
376	MIB_4674	9.0	9.0	9.0
377	MIB_4676	7.8	7.7	7.8
378	MIB_4679	7.8	7.6	7.7
379	MIB_4680	8.5	8.5	8.5
380	MIB_4682	7.2	7.1	7.2
381	MIB_4683	8.7	8.7	8.7
382	MIB_4691	8.1	8.0	8.1
383	MIB_4697	5.6	5.4	5.5
384	MIB_4699	8.6	8.7	8.7
385	MIB_4700	8.1	7.9	8.0
386	MIB_4701	6.4	6.2	6.3
387	MIB_4707	7.3	8.3	7.8
388	MIB_4712	6.9	6.7	6.8
389	MIB_4716	7.8	7.7	7.7
390	MIB_4718	8.6	8.6	8.6
391	MIB_4719	6.8	6.7	6.7

Supplementary Table S 3: SNP trait associations identified by different single and multi locus GWAS models

**GLM**					**MLM**				
S.No	SNP	Chromosome	Position	P.value	S.No	SNP	Chromosome	Position	P.value
1	S1_13264894	1	13264894	1.46E-05	1	S1_13273091	1	13273091	0.000132
2	S1_13273091	1	13273091	1.79E-05	2	S1_13264894	1	13264894	0.000167
3	S1_13274423	1	13274423	2.22E-05	3	S1_13274423	1	13274423	0.000311
4	S1_13374131	1	13374131	5.70E-05	4	S1_13424589	1	13424589	0.000475
5	S1_13365703	1	13365703	5.70E-05	5	S1_13365703	1	13365703	0.000635
6	S1_13374151	1	13374151	5.70E-05	6	S1_13374151	1	13374151	0.000635
7	S1_13898444	1	13898444	5.76E-05	7	S1_13374131	1	13374131	0.000635
8	S1_13424589	1	13424589	6.06E-05	8	S1_12742211	1	12742211	0.000821
9	S1_13316461	1	13316461	0.000117	9	S1_13316461	1	13316461	0.001485
10	S1_13333531	1	13333531	0.000117	10	S1_13333531	1	13333531	0.001485
11	S1_13323794	1	13323794	0.000117	11	S1_13323794	1	13323794	0.001485
12	S1_13541723	1	13541723	0.000147	12	S1_12737403	1	12737403	0.001936
13	S1_13541727	1	13541727	0.000147	13	S2_34796411	2	34796411	0.001204
14	S1_13541645	1	13541645	0.000147	14	S4_4571354	4	4571354	0.000892
15	S1_13776350	1	13776350	0.000151	15	S4_5135455	4	5135455	0.001716
16	S1_12742211	1	12742211	0.000169	16	S5_642108	5	642108	5.14E-05
17	S1_13542361	1	13542361	0.000262	17	S5_23257108	5	23257108	0.001079
18	S1_13542375	1	13542375	0.000262	18	S5_23279042	5	23279042	0.001079
19	S1_14099875	1	14099875	0.000264	19	S5_23256042	5	23256042	0.001079
20	S1_13956977	1	13956977	0.000302	20	S5_23257107	5	23257107	0.001079
21	S1_13563387	1	13563387	0.000392	21	S5_23279024	5	23279024	0.001079
22	S1_13567724	1	13567724	0.000452	22	S5_649114	5	649114	0.001493
23	S1_13567733	1	13567733	0.000452	23	S5_786707	5	786707	0.00175
24	S1_12737403	1	12737403	0.000474	24	S5_23287785	5	23287785	0.001787
25	S1_14102208	1	14102208	0.000646	25	S5_23303726	5	23303726	0.001787
26	S1_12704071	1	12704071	0.000747	26	S5_23307521	5	23307521	0.001787

27	S1_14060399	1	14060399	0.000799	27	S5_23310970	5	23310970	0.001787
28	S1_13541716	1	13541716	0.001794	28	S5_23312204	5	23312204	0.001866
29	S2_34796411	2	34796411	7.72E-05	29	S5_23314218	5	23314218	0.001866
30	S2_1525753	2	1525753	0.000451	30	S6_8886408	6	8886408	2.56E-05
31	S2_1525874	2	1525874	0.000451	31	S6_8886434	6	8886434	2.56E-05
32	S2_1517365	2	1517365	0.000451	32	S6_8876610	6	8876610	8.03E-05
33	S2_1531980	2	1531980	0.000669	33	S6_8851568	6	8851568	8.03E-05
34	S2_1344992	2	1344992	0.000959	34	S6_8870236	6	8870236	8.03E-05
35	S2_1550369	2	1550369	0.001441	35	S6_8876540	6	8876540	8.03E-05
36	S2_1548690	2	1548690	0.001441	36	S6_8876586	6	8876586	8.03E-05
37	S2_1553290	2	1553290	0.001441	37	S6_8876606	6	8876606	8.03E-05
38	S2_34784688	2	34784688	0.001506	38	S6_8914650	6	8914650	0.000124
39	S2_34791393	2	34791393	0.001506	39	S6_8914671	6	8914671	0.000124
40	S2_34791395	2	34791395	0.001506	40	S6_8914769	6	8914769	0.000124
41	S2_1039309	2	1039309	0.001528	41	S6_8914643	6	8914643	0.000124
42	S2_1416970	2	1416970	0.001603	42	S6_8914651	6	8914651	0.000124
43	S2_8165184	2	8165184	0.00163	43	S6_8850552	6	8850552	0.000137
44	S2_8165190	2	8165190	0.00163	44	S6_9004225	6	9004225	0.000182
45	S2_8165191	2	8165191	0.00163	45	S6_8917933	6	8917933	0.000264
46	S2_8165186	2	8165186	0.00163	46	S6_8917936	6	8917936	0.000264
47	S2_8165187	2	8165187	0.00163	47	S6_8917962	6	8917962	0.000264
48	S2_1564468	2	1564468	0.001717	48	S6_8917185	6	8917185	0.000264
49	S2_1570980	2	1570980	0.001717	49	S6_8962742	6	8962742	0.000336
50	S4_4571354	4	4571354	0.000104	50	S6_8962796	6	8962796	0.000336
51	S4_3469974	4	3469974	0.000468	51	S6_8956202	6	8956202	0.000336
52	S4_3419234	4	3419234	0.00084	52	S6_9003844	6	9003844	0.000352
53	S4_3411505	4	3411505	0.000863	53	S6_9003866	6	9003866	0.000352
54	S4_3411567	4	3411567	0.000863	54	S6_6758215	6	6758215	0.000397
55	S4_3411593	4	3411593	0.000863	55	S6_8921201	6	8921201	0.000423
56	S4_3411559	4	3411559	0.000863	56	S6_8932488	6	8932488	0.000423

57	S4_5135455	4	5135455	0.001496	57	S6_8921200	6	8921200	0.000423
58	S4_4521327	4	4521327	0.001557	58	S6_6752461	6	6752461	0.000824
59	S4_4541668	4	4541668	0.001768	59	S6_6769492	6	6769492	0.000831
60	S4_4541738	4	4541738	0.001768	60	S6_7646336	6	7646336	0.000915
61	S4_5076100	4	5076100	0.001855	61	S6_8975500	6	8975500	0.000931
62	S4_5006357	4	5006357	0.001873	62	S6_8977107	6	8977107	0.000931
63	S5_642108	5	642108	4.71E-06	63	S6_8977116	6	8977116	0.000931
64	S5_786707	5	786707	0.000242	64	S6_8977156	6	8977156	0.000931
65	S5_23256042	5	23256042	0.000279	65	S6_8977190	6	8977190	0.000931
66	S5_23257107	5	23257107	0.000279	66	S6_6823579	6	6823579	0.001205
67	S5_23279024	5	23279024	0.000279	67	S6_6769526	6	6769526	0.00122
68	S5_23257108	5	23257108	0.000279	68	S6_6769533	6	6769533	0.00122
69	S5_23279042	5	23279042	0.000279	69	S6_7630128	6	7630128	0.001321
70	S5_23287785	5	23287785	0.000506	70	S6_7614974	6	7614974	0.001321
71	S5_23303726	5	23303726	0.000506	71	S6_7626543	6	7626543	0.001321
72	S5_23307521	5	23307521	0.000506	72	S6_8990795	6	8990795	0.001355
73	S5_23310970	5	23310970	0.000506	73	S6_6819248	6	6819248	0.0015
74	S5_23408360	5	23408360	0.000525	74	S6_7647695	6	7647695	0.001582
75	S5_23417869	5	23417869	0.000525	75	S6_7647706	6	7647706	0.001582
76	S5_23426824	5	23426824	0.000525	76	S6_8977712	6	8977712	0.001772
77	S5_23433965	5	23433965	0.000525	77	S6_7709724	6	7709724	0.001833
78	S5_23436011	5	23436011	0.000525	78	S6_9028593	6	9028593	0.001833
79	S5_23443154	5	23443154	0.000525	79	S10_18115531	10	18115531	0.000622
80	S5_23467123	5	23467123	0.000525	80	S10_18127881	10	18127881	0.001102
81	S5_23497990	5	23497990	0.000525	81	S10_18145180	10	18145180	0.001102
82	S5_23504636	5	23504636	0.000525	82	S10_18387130	10	18387130	0.001351
83	S5_23508245	5	23508245	0.000525	83	S10_18393744	10	18393744	0.001351
84	S5_23509334	5	23509334	0.000525	84	S10_18851296	10	18851296	0.001654
85	S5_23434999	5	23434999	0.000525	85	S10_18870300	10	18870300	0.001654
86	S5_23448630	5	23448630	0.000525	86	S10_18441292	10	18441292	0.00166

87	S5_23463399	5	23463399	0.000525	87	S10_18402152	10	18402152	0.001803
88	S5_23504663	5	23504663	0.000525	88	S12_25336024	12	25336024	0.000585
89	S5_23509333	5	23509333	0.000525	89	S12_25336023	12	25336023	0.000585
90	S5_23314218	5	23314218	0.000564	90	S12_25343744	12	25343744	0.001566
91	S5_23312204	5	23312204	0.000564	91	S12_25343720	12	25343720	0.001566
92	S5_23240418	5	23240418	0.000577					
93	S5_23249078	5	23249078	0.000577					
94	S5_23249237	5	23249237	0.000577					
95	S5_23249119	5	23249119	0.000577					
96	S5_23249125	5	23249125	0.000577					
97	S5_23249605	5	23249605	0.000577					
98	S5_649114	5	649114	0.000857					
99	S5_966011	5	966011	0.000943					
100	S5_23386585	5	23386585	0.001015					
101	S5_23386596	5	23386596	0.001015					
102	S5_23404379	5	23404379	0.001015					
103	S6_8886408	6	8886408	1.47E-06					
104	S6_8886434	6	8886434	1.47E-06					
105	S6_8876610	6	8876610	3.69E-06					
106	S6_8851568	6	8851568	3.69E-06					
107	S6_8870236	6	8870236	3.69E-06					
108	S6_8876540	6	8876540	3.69E-06					
109	S6_8876586	6	8876586	3.69E-06					
110	S6_8876606	6	8876606	3.69E-06					
111	S6_8850552	6	8850552	4.80E-06					
112	S6_8914650	6	8914650	6.13E-06					
113	S6_8914671	6	8914671	6.13E-06					
114	S6_8914769	6	8914769	6.13E-06					
115	S6_8914643	6	8914643	6.13E-06					
116	S6_8914651	6	8914651	6.13E-06					

117	S6_9004225	6	9004225	6.88E-06
118	S6_8917933	6	8917933	1.03E-05
119	S6_8917936	6	8917936	1.03E-05
120	S6_8917962	6	8917962	1.03E-05
121	S6_8917185	6	8917185	1.03E-05
122	S6_9003844	6	9003844	1.26E-05
123	S6_9003866	6	9003866	1.26E-05
124	S6_8962742	6	8962742	1.38E-05
125	S6_8962796	6	8962796	1.38E-05
126	S6_8956202	6	8956202	1.38E-05
127	S6_8921200	6	8921200	1.54E-05
128	S6_8921201	6	8921201	1.54E-05
129	S6_8932488	6	8932488	1.54E-05
130	S6_8990795	6	8990795	2.97E-05
131	S6_8975500	6	8975500	3.23E-05
132	S6_8977107	6	8977107	3.23E-05
133	S6_8977116	6	8977116	3.23E-05
134	S6_8977156	6	8977156	3.23E-05
135	S6_8977190	6	8977190	3.23E-05
136	S6_6758215	6	6758215	3.87E-05
137	S6_9028593	6	9028593	4.01E-05
138	S6_8977712	6	8977712	4.75E-05
139	S6_9043197	6	9043197	5.11E-05
140	S6_8982135	6	8982135	5.82E-05
141	S6_8977949	6	8977949	6.99E-05
142	S6_8977972	6	8977972	6.99E-05
143	S6_6752461	6	6752461	7.36E-05
144	S6_6769492	6	6769492	7.90E-05
145	S6_7709724	6	7709724	9.21E-05
146	S6_7647695	6	7647695	0.000103

147	S6_7647706	6	7647706	0.000103
148	S6_7531094	6	7531094	0.000108
149	S6_7557807	6	7557807	0.000108
150	S6_7538495	6	7538495	0.000108
151	S6_7542726	6	7542726	0.000108
152	S6_6922808	6	6922808	0.000112
153	S6_6819248	6	6819248	0.000118
154	S6_6823579	6	6823579	0.000121
155	S6_6769526	6	6769526	0.000132
156	S6_6769533	6	6769533	0.000132
157	S6_7646336	6	7646336	0.000156
158	S6_7630128	6	7630128	0.00017
159	S6_7614974	6	7614974	0.00017
160	S6_7626543	6	7626543	0.00017
161	S6_6916461	6	6916461	0.000264
162	S6_7008351	6	7008351	0.000365
163	S6_6996522	6	6996522	0.000365
164	S6_7001492	6	7001492	0.000365
165	S6_7045328	6	7045328	0.00057
166	S6_7045355	6	7045355	0.00057
167	S6_6697070	6	6697070	0.000606
168	S6_7531433	6	7531433	0.000619
169	S6_7531437	6	7531437	0.000619
170	S6_7557905	6	7557905	0.000619
171	S6_7581809	6	7581809	0.000628
172	S6_6916223	6	6916223	0.000714
173	S6_6535236	6	6535236	0.000758
174	S6_6535237	6	6535237	0.000758
175	S6_6916163	6	6916163	0.000761
176	S6_6869009	6	6869009	0.000801

177	S6_8153078	6	8153078	0.00081
178	S6_6924356	6	6924356	0.000924
179	S6_6924359	6	6924359	0.000924
180	S6_6934785	6	6934785	0.000924
181	S6_6924352	6	6924352	0.000924
182	S6_6924355	6	6924355	0.000924
183	S6_6924358	6	6924358	0.000924
184	S6_6924361	6	6924361	0.000924
185	S6_27969065	6	27969065	0.001046
186	S6_27969545	6	27969545	0.001046
187	S6_7522397	6	7522397	0.001076
188	S6_7094512	6	7094512	0.001266
189	S6_6816739	6	6816739	0.001373
190	S6_6513819	6	6513819	0.001585

**CMLM**					**SUPER**				
S.No	SNP	Chromosome	Position	P.value	S.No	SNP	Chromosome	Position	P.value
1	S1_13273091	1	13273091	0.00027	1	S1_13264894	1	13264894	2.16E-05
2	S1_13264894	1	13264894	0.000316	2	S1_13273091	1	13273091	2.38E-05
3	S1_13274423	1	13274423	0.000587	3	S1_13274423	1	13274423	4.56E-05
4	S1_13424589	1	13424589	0.000781	4	S1_13365703	1	13365703	0.000118
5	S1_12742211	1	12742211	0.001161	5	S1_13374151	1	13374151	0.000118
6	S1_13374131	1	13374131	0.001282	6	S1_13374131	1	13374131	0.000118
7	S1_13365703	1	13365703	0.001282	7	S1_13898444	1	13898444	0.000148
8	S1_13374151	1	13374151	0.001282	8	S1_13424589	1	13424589	0.000162
9	S2_1517365	2	1517365	0.001537	9	S1_13316461	1	13316461	0.000284
10	S2_1525753	2	1525753	0.001537	10	S1_13333531	1	13333531	0.000284
11	S2_1525874	2	1525874	0.001537	11	S1_13323794	1	13323794	0.000284
12	S2_1531980	2	1531980	0.001772	12	S1_13541723	1	13541723	0.000292

13	S2_34796411	2	34796411	0.001789	13	S1_13541727	1	13541727	0.000292
14	S4_4571354	4	4571354	0.001181	14	S1_13541645	1	13541645	0.000292
15	S5_642108	5	642108	3.00E-05	15	S1_13776350	1	13776350	0.000294
16	S5_649114	5	649114	0.000907	16	S1_13956977	1	13956977	0.000401
17	S5_23257108	5	23257108	0.001069	17	S1_12742211	1	12742211	0.000506
18	S5_23279042	5	23279042	0.001069	18	S1_14099875	1	14099875	0.000551
19	S5_23256042	5	23256042	0.001069	19	S1_13563387	1	13563387	0.00076
20	S5_23257107	5	23257107	0.001069	20	S1_13542361	1	13542361	0.000829
21	S5_23279024	5	23279024	0.001069	21	S1_13542375	1	13542375	0.000829
22	S5_786707	5	786707	0.001558	22	S1_13567724	1	13567724	0.000937
23	S5_23287785	5	23287785	0.001746	23	S1_13567733	1	13567733	0.000937
24	S5_23303726	5	23303726	0.001746	24	S1_14060399	1	14060399	0.001308
25	S5_23307521	5	23307521	0.001746	25	S1_12737403	1	12737403	0.001331
26	S5_23310970	5	23310970	0.001746	26	S1_22957410	1	22957410	0.001404
27	S5_23312204	5	23312204	0.00177	27	S1_13541716	1	13541716	0.001701
28	S5_23314218	5	23314218	0.00177	28	S1_14102208	1	14102208	0.001703
29	S6_8886408	6	8886408	5.59E-05	29	S1_13584658	1	13584658	0.001818
30	S6_8886434	6	8886434	5.59E-05	30	S1_12704071	1	12704071	0.001966
31	S6_8851568	6	8851568	0.000164	31	S2_34796411	2	34796411	0.000224
32	S6_8870236	6	8870236	0.000164	32	S2_1344992	2	1344992	0.000701
33	S6_8876540	6	8876540	0.000164	33	S2_1517365	2	1517365	0.001077
34	S6_8876586	6	8876586	0.000164	34	S2_1525753	2	1525753	0.001077
35	S6_8876606	6	8876606	0.000164	35	S2_1525874	2	1525874	0.001077
36	S6_8876610	6	8876610	0.000164	36	S2_1531980	2	1531980	0.00123
37	S6_8850552	6	8850552	0.000222	37	S2_35179606	2	35179606	0.001561
38	S6_8914643	6	8914643	0.00027	38	S2_1416970	2	1416970	0.001649
39	S6_8914651	6	8914651	0.00027	39	S2_34784688	2	34784688	0.001664
40	S6_8914650	6	8914650	0.00027	40	S2_34791393	2	34791393	0.001664
41	S6_8914671	6	8914671	0.00027	41	S2_34791395	2	34791395	0.001664
42	S6_8914769	6	8914769	0.00027	42	S2_29638136	2	29638136	0.00184

43	S6_9004225	6	9004225	0.0004	43	S2_29640532	2	29640532	0.00184
44	S6_6758215	6	6758215	0.000474	44	S4_4571354	4	4571354	0.000149
45	S6_8917933	6	8917933	0.000572	45	S4_3469974	4	3469974	0.00048
46	S6_8917936	6	8917936	0.000572	46	S4_5135455	4	5135455	0.000965
47	S6_8917962	6	8917962	0.000572	47	S4_3411559	4	3411559	0.001148
48	S6_8917185	6	8917185	0.000572	48	S4_3411505	4	3411505	0.001148
49	S6_8962742	6	8962742	0.000724	49	S4_3411567	4	3411567	0.001148
50	S6_8962796	6	8962796	0.000724	50	S4_3411593	4	3411593	0.001148
51	S6_8956202	6	8956202	0.000724	51	S4_3419234	4	3419234	0.001166
52	S6_9003844	6	9003844	0.000733	52	S4_5076100	4	5076100	0.001489
53	S6_9003866	6	9003866	0.000733	53	S4_4521327	4	4521327	0.001589
54	S6_6752461	6	6752461	0.000839	54	S4_4822959	4	4822959	0.001713
55	S6_8921201	6	8921201	0.000916	55	S5_23256042	5	23256042	0.000333
56	S6_8932488	6	8932488	0.000916	56	S5_23257107	5	23257107	0.000333
57	S6_8921200	6	8921200	0.000916	57	S5_23279024	5	23279024	0.000333
58	S6_6769492	6	6769492	0.000984	58	S5_23257108	5	23257108	0.000333
59	S6_6823579	6	6823579	0.001265	59	S5_23279042	5	23279042	0.000333
60	S6_7646336	6	7646336	0.001544	60	S5_642108	5	642108	0.000406
61	S6_6819248	6	6819248	0.001553	61	S5_23314218	5	23314218	0.000438
62	S6_6769526	6	6769526	0.001727	62	S5_23312204	5	23312204	0.000438
63	S6_6769533	6	6769533	0.001727	63	S5_23287785	5	23287785	0.000507
64	S6_8975500	6	8975500	0.001852	64	S5_23303726	5	23303726	0.000507
65	S6_8977107	6	8977107	0.001852	65	S5_23307521	5	23307521	0.000507
66	S6_8977116	6	8977116	0.001852	66	S5_23310970	5	23310970	0.000507
67	S6_8977156	6	8977156	0.001852	67	S5_23434999	5	23434999	0.000513
68	S6_8977190	6	8977190	0.001852	68	S5_23448630	5	23448630	0.000513
69	S10_18115531	10	18115531	0.000757	69	S5_23463399	5	23463399	0.000513
70	S10_18145180	10	18145180	0.00119	70	S5_23504663	5	23504663	0.000513
71	S10_18127881	10	18127881	0.00119	71	S5_23509333	5	23509333	0.000513
72	S10_18387130	10	18387130	0.001451	72	S5_23408360	5	23408360	0.000513

73	S10_18393744	10	18393744	0.001451
74	S10_18851296	10	18851296	0.001628
75	S10_18870300	10	18870300	0.001628
76	S10_18441292	10	18441292	0.001889
77	S12_25336023	12	25336023	0.000607
78	S12_25336024	12	25336024	0.000607
79	S12_25343744	12	25343744	0.001283
80	S12_25343720	12	25343720	0.001283

73	S5_23417869	5	23417869	0.000513
74	S5_23426824	5	23426824	0.000513
75	S5_23433965	5	23433965	0.000513
76	S5_23436011	5	23436011	0.000513
77	S5_23443154	5	23443154	0.000513
78	S5_23467123	5	23467123	0.000513
79	S5_23497990	5	23497990	0.000513
80	S5_23504636	5	23504636	0.000513
81	S5_23508245	5	23508245	0.000513
82	S5_23509334	5	23509334	0.000513
83	S5_23249119	5	23249119	0.000653
84	S5_23249125	5	23249125	0.000653
85	S5_23249605	5	23249605	0.000653
86	S5_23240418	5	23240418	0.000653
87	S5_23249078	5	23249078	0.000653
88	S5_23249237	5	23249237	0.000653
89	S5_23386585	5	23386585	0.00066
90	S5_23386596	5	23386596	0.00066
91	S5_23404379	5	23404379	0.00066
92	S5_25071334	5	25071334	0.00194
93	S5_25044791	5	25044791	0.00194
94	S6_8886408	6	8886408	1.10E-06
95	S6_8886434	6	8886434	1.10E-06
96	S6_8876610	6	8876610	2.67E-06
97	S6_8851568	6	8851568	2.67E-06
98	S6_8870236	6	8870236	2.67E-06
99	S6_8876540	6	8876540	2.67E-06
100	S6_8876586	6	8876586	2.67E-06
101	S6_8876606	6	8876606	2.67E-06
102	S6_8850552	6	8850552	3.52E-06

103	S6_9004225	6	9004225	4.47E-06
104	S6_8914650	6	8914650	4.63E-06
105	S6_8914671	6	8914671	4.63E-06
106	S6_8914769	6	8914769	4.63E-06
107	S6_8914643	6	8914643	4.63E-06
108	S6_8914651	6	8914651	4.63E-06
109	S6_9003844	6	9003844	8.14E-06
110	S6_9003866	6	9003866	8.14E-06
111	S6_8962742	6	8962742	9.40E-06
112	S6_8962796	6	8962796	9.40E-06
113	S6_8956202	6	8956202	9.40E-06
114	S6_8917185	6	8917185	9.92E-06
115	S6_8917933	6	8917933	9.92E-06
116	S6_8917936	6	8917936	9.92E-06
117	S6_8917962	6	8917962	9.92E-06
118	S6_8921201	6	8921201	1.48E-05
119	S6_8932488	6	8932488	1.48E-05
120	S6_8921200	6	8921200	1.48E-05
121	S6_9028593	6	9028593	1.77E-05
122	S6_8977156	6	8977156	2.16E-05
123	S6_8977190	6	8977190	2.16E-05
124	S6_8975500	6	8975500	2.16E-05
125	S6_8977107	6	8977107	2.16E-05
126	S6_8977116	6	8977116	2.16E-05
127	S6_8990795	6	8990795	2.18E-05
128	S6_9043197	6	9043197	2.27E-05
129	S6_8977712	6	8977712	3.07E-05
130	S6_8982135	6	8982135	3.43E-05
131	S6_8977949	6	8977949	4.15E-05
132	S6_8977972	6	8977972	4.15E-05

133	S6_6758215	6	6758215	9.19E-05
134	S6_7646336	6	7646336	0.000119
135	S6_7647706	6	7647706	0.000132
136	S6_7647695	6	7647695	0.000132
137	S6_6769533	6	6769533	0.000143
138	S6_6769526	6	6769526	0.000143
139	S6_6769492	6	6769492	0.000151
140	S6_7630128	6	7630128	0.000158
141	S6_7614974	6	7614974	0.000158
142	S6_7626543	6	7626543	0.000158
143	S6_7709724	6	7709724	0.000177
144	S6_7538495	6	7538495	0.000205
145	S6_7542726	6	7542726	0.000205
146	S6_7531094	6	7531094	0.000205
147	S6_7557807	6	7557807	0.000205
148	S6_6922808	6	6922808	0.000595
149	S6_6916461	6	6916461	0.001228
150	S6_6535237	6	6535237	0.00124
151	S6_6535236	6	6535236	0.00124
152	S6_8153078	6	8153078	0.001248
153	S6_6697070	6	6697070	0.001742
154	S7_10995384	7	10995384	0.001918
155	S10_18127881	10	18127881	0.001606
156	S10_18145180	10	18145180	0.001606
157	S11_17207110	11	17207110	0.001643
158	S11_17207113	11	17207113	0.001643
159	S12_25336023	12	25336023	0.000843
160	S12_25336024	12	25336024	0.000843
161	S12_25343720	12	25343720	0.001685
162	S12_25343744	12	25343744	0.001685

Farm CPU					MLMM				
S.No	SNP	Chromosome	Position	P.value	S.No	SNP	Chromosome	Position	P.value
1	S1_13273091	1	13273091	0.000205	1	S1_13264894	1	13264894	0.000112
2	S1_13274423	1	13274423	0.000446	2	S1_13273091	1	13273091	0.000115
3	S1_13374131	1	13374131	0.000984	3	S1_13274423	1	13274423	0.000217
4	S1_13365703	1	13365703	0.000984	4	S1_13424589	1	13424589	0.000348
5	S1_13374151	1	13374151	0.000984	5	S1_13365703	1	13365703	0.000512
6	S1_13424589	1	13424589	0.001092	6	S1_13374131	1	13374131	0.000512
7	S1_13898444	1	13898444	0.001108	7	S1_13374151	1	13374151	0.000512
8	S1_12742211	1	12742211	0.001715	8	S1_12742211	1	12742211	0.000658
9	S1_13323794	1	13323794	0.001732	9	S1_13316461	1	13316461	0.001251
10	S1_13316461	1	13316461	0.001732	10	S1_13323794	1	13323794	0.001251
11	S1_13333531	1	13333531	0.001732	11	S1_13333531	1	13333531	0.001251
12	S1_13541723	1	13541723	0.002529	12	S1_12737403	1	12737403	0.001632
13	S1_13541727	1	13541727	0.002529	13	S1_13898444	1	13898444	0.001692
14	S1_13541645	1	13541645	0.002529	14	S1_12704071	1	12704071	0.002559
15	S1_13776350	1	13776350	0.002552	15	S1_13541645	1	13541645	0.002719
16	S1_13956977	1	13956977	0.00288	16	S1_13541723	1	13541723	0.002719
17	S1_14099875	1	14099875	0.003902	17	S1_13541727	1	13541727	0.002719
18	S1_12737403	1	12737403	0.003939	18	S1_1265155	1	1265155	0.002824
19	S1_13542361	1	13542361	0.004021	19	S1_13956977	1	13956977	0.003332
20	S1_13542375	1	13542375	0.004021	20	S1_13776350	1	13776350	0.003391
21	S2_34796411	2	34796411	0.00059	21	S1_1963118	1	1963118	0.003447
22	S2_1525753	2	1525753	0.00147	22	S1_1963129	1	1963129	0.003447
23	S2_1525874	2	1525874	0.00147	23	S1_2271313	1	2271313	0.003656
24	S2_1517365	2	1517365	0.00147	24	S1_12650673	1	12650673	0.004319
25	S2_1531980	2	1531980	0.002093	25	S1_13542361	1	13542361	0.005064
26	S2_1344992	2	1344992	0.002499	26	S1_13542375	1	13542375	0.005064
27	S2_34784688	2	34784688	0.002872	27	S2_34796411	2	34796411	0.00073
28	S2_34791393	2	34791393	0.002872	28	S2_1517365	2	1517365	0.001381

29	S2_34791395	2	34791395	0.002872	29	S2_1525753	2	1525753	0.001381
30	S2_1416970	2	1416970	0.003939	30	S2_1525874	2	1525874	0.001381
31	S2_8165184	2	8165184	0.004014	31	S2_1531980	2	1531980	0.00167
32	S2_8165190	2	8165190	0.004014	32	S2_1344992	2	1344992	0.002302
33	S2_8165191	2	8165191	0.004014	33	S2_1039309	2	1039309	0.003138
34	S2_8165186	2	8165186	0.004014	34	S2_1548690	2	1548690	0.003775
35	S2_8165187	2	8165187	0.004014	35	S2_1550369	2	1550369	0.003775
36	S2_1550369	2	1550369	0.004058	36	S2_1553290	2	1553290	0.003775
37	S2_1548690	2	1548690	0.004058	37	S2_1570980	2	1570980	0.004178
38	S2_1553290	2	1553290	0.004058	38	S2_1564468	2	1564468	0.004178
39	S2_1570980	2	1570980	0.0048	39	S2_1416970	2	1416970	0.004188
40	S2_1564468	2	1564468	0.0048	40	S4_4571354	4	4571354	0.000831
41	S4_4571354	4	4571354	0.000391	41	S4_5135455	4	5135455	0.001575
42	S4_3469974	4	3469974	0.001551	42	S4_5076100	4	5076100	0.002623
43	S4_3419234	4	3419234	0.002479	43	S4_4822959	4	4822959	0.00382
44	S4_3411505	4	3411505	0.002492	44	S5_642108	5	642108	1.39E-05
45	S4_3411567	4	3411567	0.002492	45	S5_649114	5	649114	0.000834
46	S4_3411593	4	3411593	0.002492	46	S5_23256042	5	23256042	0.000914
47	S4_3411559	4	3411559	0.002492	47	S5_23257107	5	23257107	0.000914
48	S4_5135455	4	5135455	0.0034	48	S5_23257108	5	23257108	0.000914
49	S4_5076100	4	5076100	0.004232	49	S5_23279024	5	23279024	0.000914
50	S4_4937112	4	4937112	0.004951	50	S5_23279042	5	23279042	0.000914
51	S4_4937146	4	4937146	0.004951	51	S5_786707	5	786707	0.00104
52	S5_23256042	5	23256042	0.000598	52	S5_23287785	5	23287785	0.001568
53	S5_23257107	5	23257107	0.000598	53	S5_23303726	5	23303726	0.001568
54	S5_23279024	5	23279024	0.000598	54	S5_23307521	5	23307521	0.001568
55	S5_23257108	5	23257108	0.000598	55	S5_23310970	5	23310970	0.001568
56	S5_23279042	5	23279042	0.000598	56	S5_23312204	5	23312204	0.001614
57	S5_23287785	5	23287785	0.001032	57	S5_23314218	5	23314218	0.001614
58	S5_23303726	5	23303726	0.001032	58	S5_23240418	5	23240418	0.001863

59	S5_23307521	5	23307521	0.001032	59	S5_23249078	5	23249078	0.001863
60	S5_23310970	5	23310970	0.001032	60	S5_23249119	5	23249119	0.001863
61	S5_23408360	5	23408360	0.001067	61	S5_23249125	5	23249125	0.001863
62	S5_23417869	5	23417869	0.001067	62	S5_23249237	5	23249237	0.001863
63	S5_23426824	5	23426824	0.001067	63	S5_23249605	5	23249605	0.001863
64	S5_23433965	5	23433965	0.001067	64	S5_23408360	5	23408360	0.001943
65	S5_23436011	5	23436011	0.001067	65	S5_23417869	5	23417869	0.001943
66	S5_23443154	5	23443154	0.001067	66	S5_23426824	5	23426824	0.001943
67	S5_23467123	5	23467123	0.001067	67	S5_23433965	5	23433965	0.001943
68	S5_23497990	5	23497990	0.001067	68	S5_23434999	5	23434999	0.001943
69	S5_23504636	5	23504636	0.001067	69	S5_23436011	5	23436011	0.001943
70	S5_23508245	5	23508245	0.001067	70	S5_23443154	5	23443154	0.001943
71	S5_23509334	5	23509334	0.001067	71	S5_23448630	5	23448630	0.001943
72	S5_23434999	5	23434999	0.001067	72	S5_23463399	5	23463399	0.001943
73	S5_23448630	5	23448630	0.001067	73	S5_23467123	5	23467123	0.001943
74	S5_23463399	5	23463399	0.001067	74	S5_23497990	5	23497990	0.001943
75	S5_23504663	5	23504663	0.001067	75	S5_23504636	5	23504636	0.001943
76	S5_23509333	5	23509333	0.001067	76	S5_23504663	5	23504663	0.001943
77	S5_23314218	5	23314218	0.001168	77	S5_23508245	5	23508245	0.001943
78	S5_23312204	5	23312204	0.001168	78	S5_23509333	5	23509333	0.001943
79	S5_23249119	5	23249119	0.001177	79	S5_23509334	5	23509334	0.001943
80	S5_23249125	5	23249125	0.001177	80	S5_23386585	5	23386585	0.00324
81	S5_23249605	5	23249605	0.001177	81	S5_23386596	5	23386596	0.00324
82	S5_23240418	5	23240418	0.001177	82	S5_23404379	5	23404379	0.00324
83	S5_23249078	5	23249078	0.001177	83	S5_966011	5	966011	0.004407
84	S5_23249237	5	23249237	0.001177	84	S6_8886408	6	8886408	2.33E-05
85	S5_786707	5	786707	0.001774	85	S6_8886434	6	8886434	2.33E-05
86	S5_966011	5	966011	0.001831	86	S6_8851568	6	8851568	7.85E-05
87	S5_23386585	5	23386585	0.00199	87	S6_8870236	6	8870236	7.85E-05
88	S5_23386596	5	23386596	0.00199	88	S6_8876540	6	8876540	7.85E-05

89	S5_23404379	5	23404379	0.00199	89	S6_8876586	6	8876586	7.85E-05
90	S5_1080429	5	1080429	0.004782	90	S6_8876606	6	8876606	7.85E-05
91	S6_8886408	6	8886408	1.16E-05	91	S6_8876610	6	8876610	7.85E-05
92	S6_8886434	6	8886434	1.16E-05	92	S6_8850552	6	8850552	0.000107
93	S6_8851568	6	8851568	2.73E-05	93	S6_8914643	6	8914643	0.000137
94	S6_8870236	6	8870236	2.73E-05	94	S6_8914650	6	8914650	0.000137
95	S6_8876540	6	8876540	2.73E-05	95	S6_8914651	6	8914651	0.000137
96	S6_8876586	6	8876586	2.73E-05	96	S6_8914671	6	8914671	0.000137
97	S6_8876606	6	8876606	2.73E-05	97	S6_8914769	6	8914769	0.000137
98	S6_8876610	6	8876610	2.73E-05	98	S6_9004225	6	9004225	0.000214
99	S6_8850552	6	8850552	3.47E-05	99	S6_6758215	6	6758215	0.000291
100	S6_8914643	6	8914643	4.38E-05	100	S6_8917185	6	8917185	0.000303
101	S6_8914651	6	8914651	4.38E-05	101	S6_8917933	6	8917933	0.000303
102	S6_8914650	6	8914650	4.38E-05	102	S6_8917936	6	8917936	0.000303
103	S6_8914671	6	8914671	4.38E-05	103	S6_8917962	6	8917962	0.000303
104	S6_8914769	6	8914769	4.38E-05	104	S6_8956202	6	8956202	0.00039
105	S6_9004225	6	9004225	5.08E-05	105	S6_8962742	6	8962742	0.00039
106	S6_8917185	6	8917185	7.10E-05	106	S6_8962796	6	8962796	0.00039
107	S6_8917933	6	8917933	7.10E-05	107	S6_9003844	6	9003844	0.000412
108	S6_8917936	6	8917936	7.10E-05	108	S6_9003866	6	9003866	0.000412
109	S6_8917962	6	8917962	7.10E-05	109	S6_8921200	6	8921200	0.0005
110	S6_9003844	6	9003844	8.72E-05	110	S6_8921201	6	8921201	0.0005
111	S6_9003866	6	9003866	8.72E-05	111	S6_8932488	6	8932488	0.0005
112	S6_8956202	6	8956202	9.28E-05	112	S6_6752461	6	6752461	0.000506
113	S6_8962742	6	8962742	9.28E-05	113	S6_6769492	6	6769492	0.00066
114	S6_8962796	6	8962796	9.28E-05	114	S6_6823579	6	6823579	0.000775
115	S6_8921200	6	8921200	0.000102	115	S6_6819248	6	6819248	0.000983
116	S6_8921201	6	8921201	0.000102	116	S6_7646336	6	7646336	0.001025
117	S6_8932488	6	8932488	0.000102	117	S6_8975500	6	8975500	0.001114
118	S6_8990795	6	8990795	0.00019	118	S6_8977107	6	8977107	0.001114

119	S6_8977156	6	8977156	0.000201	119	S6_8977116	6	8977116	0.001114
120	S6_8977190	6	8977190	0.000201	120	S6_8977156	6	8977156	0.001114
121	S6_8975500	6	8975500	0.000201	121	S6_8977190	6	8977190	0.001114
122	S6_8977107	6	8977107	0.000201	122	S6_6769533	6	6769533	0.00121
123	S6_8977116	6	8977116	0.000201	123	S6_6769526	6	6769526	0.00121
124	S6_9028593	6	9028593	0.000251	124	S6_7647695	6	7647695	0.001433
125	S6_8977712	6	8977712	0.000292	125	S6_7647706	6	7647706	0.001433
126	S6_9043197	6	9043197	0.000311	126	S6_8990795	6	8990795	0.001487
127	S6_8982135	6	8982135	0.00035	127	S6_7614974	6	7614974	0.001496
128	S6_8977972	6	8977972	0.000413	128	S6_7626543	6	7626543	0.001496
129	S6_8977949	6	8977949	0.000413	129	S6_7630128	6	7630128	0.001496
130	S6_6752461	6	6752461	0.000557	130	S6_7709724	6	7709724	0.001535
131	S6_6769492	6	6769492	0.000605	131	S6_27969065	6	27969065	0.001852
132	S6_6819248	6	6819248	0.000852	132	S6_27969545	6	27969545	0.001852
133	S6_6823579	6	6823579	0.000868	133	S6_8977712	6	8977712	0.001962
134	S6_6922808	6	6922808	0.00092	134	S6_9028593	6	9028593	0.002109
135	S6_7709724	6	7709724	0.001029	135	S6_8982135	6	8982135	0.002335
136	S6_6769533	6	6769533	0.001072	136	S6_8977949	6	8977949	0.002451
137	S6_6769526	6	6769526	0.001072	137	S6_8977972	6	8977972	0.002451
138	S6_7647706	6	7647706	0.001098	138	S6_6922808	6	6922808	0.002494
139	S6_7647695	6	7647695	0.001098	139	S6_9043197	6	9043197	0.002688
140	S6_7538495	6	7538495	0.00118	140	S6_7531094	6	7531094	0.002834
141	S6_7542726	6	7542726	0.00118	141	S6_7538495	6	7538495	0.002834
142	S6_7531094	6	7531094	0.00118	142	S6_7542726	6	7542726	0.002834
143	S6_7557807	6	7557807	0.00118	143	S6_7557807	6	7557807	0.002834
144	S6_7646336	6	7646336	0.001387	144	S6_6916461	6	6916461	0.004863
145	S6_7614974	6	7614974	0.001493	145	S10_18115531	10	18115531	0.000456
146	S6_7626543	6	7626543	0.001493	146	S10_18127881	10	18127881	0.000888
147	S6_7630128	6	7630128	0.001493	147	S10_18145180	10	18145180	0.000888
148	S6_6916461	6	6916461	0.001916	148	S10_18393744	10	18393744	0.000962

149	S6_6513819	6	6513819	0.002159	149	S10_18387130	10	18387130	0.000962
150	S6_6514012	6	6514012	0.002159	150	S10_18441292	10	18441292	0.001283
151	S6_7045328	6	7045328	0.002197	151	S10_18851296	10	18851296	0.001283
152	S6_7045355	6	7045355	0.002197	152	S10_18870300	10	18870300	0.001283
153	S6_27969065	6	27969065	0.002493	153	S10_18402152	10	18402152	0.001507
154	S6_27969545	6	27969545	0.002493	154	S10_18897171	10	18897171	0.001764
155	S6_6996522	6	6996522	0.002525	155	S10_18898657	10	18898657	0.001764
156	S6_7001492	6	7001492	0.002525	156	S10_18416827	10	18416827	0.002831
157	S6_7008351	6	7008351	0.002525	157	S10_18417432	10	18417432	0.002831
158	S6_7531433	6	7531433	0.003008	158	S10_18421895	10	18421895	0.002831
159	S6_7531437	6	7531437	0.003008	159	S10_18422007	10	18422007	0.002831
160	S6_7557905	6	7557905	0.003008	160	S10_18422024	10	18422024	0.002831
161	S6_7581809	6	7581809	0.003026	161	S10_18432420	10	18432420	0.002831
162	S6_6697070	6	6697070	0.003737	162	S10_18446898	10	18446898	0.003701
163	S6_6916223	6	6916223	0.00403	163	S10_14796704	10	14796704	0.004618
164	S6_6916163	6	6916163	0.004215	164	S12_25336023	12	25336023	0.000437
165	S6_6535236	6	6535236	0.004266	165	S12_25336024	12	25336024	0.000437
166	S6_6535237	6	6535237	0.004266	166	S12_25343720	12	25343720	0.001229
167	S6_6869009	6	6869009	0.004267	167	S12_25343744	12	25343744	0.001229
168	S6_6816739	6	6816739	0.004398					
169	S6_8153078	6	8153078	0.004465					
170	S6_7522397	6	7522397	0.004538					
171	S6_5759360	6	5759360	0.004568					
172	S6_6924352	6	6924352	0.005003					
173	S6_6924355	6	6924355	0.005003					
174	S6_6924358	6	6924358	0.005003					
175	S6_6924361	6	6924361	0.005003					
176	S6_6924356	6	6924356	0.005003					
177	S6_6924359	6	6924359	0.005003					
178	S6_6934785	6	6934785	0.005003					

179	S7_10995384	7	10995384	0.001243					
180	S7_19462828	7	19462828	0.003362					
181	S7_19463575	7	19463575	0.003362					
182	S7_19478033	7	19478033	0.003585					
183	S7_19477481	7	19477481	0.004478					
184	S10_14796704	10	14796704	0.001675					
185	S10_18115531	10	18115531	0.001865					
186	S10_18387130	10	18387130	0.003443					
187	S10_18393744	10	18393744	0.003443					
188	S10_18145180	10	18145180	0.003477					
189	S10_18127881	10	18127881	0.003477					
190	S10_14780779	10	14780779	0.004864					

Supplementary Table S 4: List of candidate genes (defense related genes) identified in the associated QTN regions

S. No.	Gene Locus ID	Chromosome	SNPs	Description or Putative Function (RAP-DB annotation)
1	LOC_Os01g22640	1	S1_12737403	GDSL-like lipase/acylhydrolase, putative, expressed
2	LOC_Os01g22660	1	S1_12742211	GDSL-like lipase/acylhydrolase, putative, expressed
3	LOC_Os01g23610	1	S1_13273091, S1_13274423	dihydrolipoyl dehydrogenase, putative, expressed
4	LOC_Os01g23670	1	S1_13316461	retrotransposon protein, putative, unclassified
5	LOC_Os01g23680	1	S1_13323794S1_13333531	rossmann fold nucleotide-binding protein involved in DNA uptake, putative, expressed
6	LOC_Os01g23780	1	S1_13365703, S1_13374131, S1_13374151	OsMADS95 - MADS-box family gene with M-beta type-box, expressed
7	LOC_Os01g23850	1	S1_13424589	transposon protein, putative, CACTA, En/Spm sub-class, expressed
8	LOC_Os01g24030	1	S1_13541645, S1_13541723, S1_13541727, S1_13542361, S1_13542375	AMP-binding enzyme, putative, expressed

9	LOC_Os01g24430	1	S1_13776350,	expressed protein
10	LOC_Os01g24690	1	S1_13898444	60S ribosomal protein L23A, putative, expressed, response to abiotic stimulus, response to stress
11	LOC_Os01g24790	1	S1_13956977	transferase family protein, putative, expressed
12	LOC_Os01g25000	1	S1_14099875	retrotransposon protein, putative, unclassified, expressed
13	LOC_Os02g03330	2	S2_1344992	expressed protein
14	LOC_Os02g03480	2	S2_1416970	THION24 - Plant thionin family protein precursor, expressed
15	LOC_Os02g03660	2	S2_1517365, S2_1525753, S2_1525874	OsFBDUF12 - F-box and DUF domain containing protein, expressed
16	LOC_Os02g03670	2	S2_1531980	expressed protein
17	LOC_Os02g03700	2	S2_1548690, S2_1550369, S2_1553290	expressed protein
18	LOC_Os02g03730	2	S2_1564468,	SWIB/MDM2 domain containing protein, expressed
19	LOC_Os02g03750	2	S2_1570980	polygalacturonase, putative, expressed
20	LOC_Os02g14760	2	S2_8165184, S2_8165186, S2_8165187, S2_8165190, S2_8165191	CID11, putative, expressed
21	LOC_Os02g56740	2	S2_34784688, S2_34791393, S2_34791395	translation initiation factor eIF-2B subunit epsilon, putative, expressed
22	LOC_Os02g56750	2	S2_34796411	OsFBX65 - F-box domain containing protein, expressed
23	LOC_Os04g06520	4	S4_3411505, S4_3411559, S4_3411567, S4_3411593, S4_3419234,	expressed protein
24	LOC_Os04g06590	4	S4_3469974	expressed protein
25	LOC_Os04g08470	4	S4_4571354,	OsFBX116 - F-box domain containing protein, expressed
26	LOC_Os04g09284	4	S4_4937112, S4_4937146	retrotransposon protein, putative, Ty3-gypsy subclass, expressed
27	LOC_Os04g09580	4	S4_5076100, S4_5135455	expressed protein
28	LOC_Os05g02400	5	S5_786707	RNA recognition motif containing protein, expressed

29	LOC_Os05g02700	5	S5_966011	expressed protein
30	LOC_Os05g02940	5	S5_1080429	calcium-transporting ATPase 2, endoplasmic reticulum-type, putative, expressed
31	LOC_Os05g39580	5	S5_23240418	bet v I allergen family protein, putative, expressed
32	LOC_Os05g39590	5	S5_23249078, S5_23249119, S5_23249125, S5_23249237, S5_23249605	AP2 domain containing protein, expressed
33	LOC_Os05g39610	5	S5_23256042, S5_23257107, S5_23257108,	exo70 exocyst complex subunit domain containing protein, expressed
34	LOC_Os05g39650	5	S5_23287785, S5_23279024	phytanoyl-CoA dioxygenase, putative, expressed
35	LOC_Os05g39700	5	S5_23303726,	hypothetical protein
36	LOC_Os05g39710	5	S5_23307521,	expressed protein
37	LOC_Os05g39720	5	S5_23310970, S5_23312204, S5_23314218	WRKY70, expressed
38	LOC_Os05g39800	5	S5_23386585, S5_23386596	NFD4, putative, expressed
39	LOC_Os05g39840	5	S5_23404379, S5_23408360, S5_23417869	expressed protein
40	LOC_Os05g39860	5	S5_23426824,	expressed protein
41	LOC_Os05g39870	5	S5_23433965	CAMK_KIN1/SNF1/Nim1_like.24 - CAMK includes calcium/calmodulin depedent protein kinases, expressed
42	LOC_Os05g39880	5	S5_23434999, S5_23436011	expressed protein
43	LOC_Os05g39900	5	S5_23443154, S5_23448630	CBL-interacting serine/threonine-protein kinase 15, putative, expressed
44	LOC_Os05g39930	5	S5_23463399, S5_23467123	spotted leaf 11, putative, expressed
45	LOC_Os05g40010	5	S5_23497990, S5_23504636, S5_23504663, S5_23508245, S5_23509333, S5_23509334	LTPL17 - Protease inhibitor/seed storage/LTP family protein precursor, expressed
46	LOC_Os06g46149	6	S6_27969065,S6_27969545	serine/arginine repetitive matrix protein 1, putative, expressed
47	LOC_Os06g11010	6	S6_5759360	eukaryotic aspartyl protease domain containing protein, expressed
48	LOC_Os06g12160.1	6	S6_6513819, S6_6514012	AAA-type ATPase family protein, putative, expressed

49	LOC_Os06g12190.1	6	S6_6535236, S6_6535237	glutaredoxin, putative, expressed
50	LOC_Os06g12360.1	6	S6_6697070	pentatricopeptide, putative, expressed
51	LOC_Os06g12450.1	6	S6_6752461	soluble starch synthase 2-3, chloroplast precursor, putative, expressed
52	LOC_Os06g12470	6	S6_6769492, S6_6769526, S6_6769533	retrotransposon protein, putative, Ty3-gypsy subclass, expressed
53	LOC_Os06g12580.1	6	S6_6816739, S6_6819248	pro-resilin precursor, putative, expressed
54	LOC_Os06g12590.1	6	S6_6823579	protein kinase, putative, expressed
55	LOC_Os06g12610.1	6	S6_6869009	auxin efflux carrier component, putative, expressed
56	LOC_Os06g12660.1	6	S6_6916163, S6_6916223, S6_6916461	NHL repeat-containing protein, putative, expressed
57	LOC_Os06g12680.1	6	S6_6922808, S6_6924352, S6_6924355, S6_6924356, S6_6924358, S6_6924359, S6_6924361	RING-H2 finger protein, putative, expressed
58	LOC_Os06g12690	6	S6_6934785	DCN1-like protein 2, putative, expressed
59	LOC_Os06g12810.1	6	S6_6996522, S6_7001492	proteins of unknown function domain containing protein, expressed
60	LOC_Os06g12820.1	6	S6_7008351	proteins of unknown function domain containing protein, expressed
61	LOC_Os06g12870.1	6	S6_7045328, S6_7045355	leaf senescence related protein, putative, expressed
62	LOC_Os06g13590	6	S6_7522397	PE-PGRS family protein, putative, expressed
63	LOC_Os06g13600.1	6	S6_7531094, S6_7531433, S6_7531437, S6_7538495	HEAT repeat family protein, putative, expressed
64	LOC_Os06g13610	6	S6_7542726	expressed protein
65	LOC_Os06g13640	6	S6_7557807, S6_7557905	expressed protein
66	LOC_Os06g13660	6	S6_7581809	alanyl-tRNA synthetase, putative, expressed
67	LOC_Os06g13740	6	S6_7614974	transposon protein, putative, unclassified, expressed
68	LOC_Os06g13760.1	6	S6_7626543, S6_7630128	glycosyl transferase 8 domain containing protein, putative, expressed
69	LOC_Os06g13800	6	S6_7646336, S6_7647695, S6_7647706	expressed protein

70	LOC_Os06g13870.1	6	S6_7709724	U-box protein CMPG1, putative, expressed
71	LOC_Os06g14510.3	6	S6_8153078	glucose-6-phosphate isomerase, putative, expressed
72	LOC_Os06g15630	6	S6_8850552, S6_8851568	retrotransposon protein, putative, unclassified, expressed
73	LOC_Os06g15660	6	S6_8870236	retrotransposon protein, putative, unclassified, expressed
74	LOC_Os06g15680.1	6	S6_8876540, S6_8876586, S6_8876606, S6_8876610	cytochrome P450 71A6, putative, expressed
75	LOC_Os06g15700	6	S6_8886408, S6_8886434	expressed protein
76	LOC_Os06g15730	6	S6_8914643, S6_8914650, S6_8914651, S6_8914671, S6_8914769, S6_8917185, S6_8917933, S6_8917936, S6_8917962, S6_8921200, S6_8921201	expressed protein
77	LOC_Os06g15750	6	S6_8932488	NB-ARC domain containing protein, expressed
78	LOC_Os06g15760.1	6	S6_8956202	eukaryotic aspartyl protease domain containing protein, expressed
79	LOC_Os06g15779.1	6	S6_8962742, S6_8962796	aluminum-activated malate transporter, putative, expressed
80	LOC_Os06g15810.1	6	S6_8975500	integral membrane protein, putative, expressed
81	LOC_Os06g15820.1	6	S6_8977107, S6_8977116, S6_8977156, S6_8977190, S6_8977712, S6_8977949, S6_8977972, S6_8982135	NHL repeat-containing protein, putative, expressed
82	LOC_Os06g15840	6	S6_8990795	transposon protein, putative, CACTA, En/Spm sub-class, expressed
83	LOC_Os06g15850	6	S6_9003844, S6_9003866, S6_9004225	transposon protein, putative, CACTA, En/Spm sub-class, expressed
84	LOC_Os06g15910	6	S6_9028593, S6_9043197	potassium transporter, putative, expressed
85	LOC_Os07g18600	7	S7_10995384	OsFBL37 - F-box domain and LRR containing protein, expressed
86	LOC_Os07g32630.1	7	S7_19462828, S7_19463575,	UDP-glucoronosyl and UDP-glucosyl transferase

				domain containing protein, expressed
87	LOC_Os07g32650	7	S7_19477481, S7_19478033	retrotransposon protein, putative, unclassified, expressed
88	LOC_Os10g28410	10	S10_14780779, S10_14796704	GA19388-PA, putative, expressed
89	LOC_Os10g33980	10	S10_18115531, S10_18127881,	thiamine pyrophosphate enzyme, N-terminal TPP binding domain containing protein, expressed
90	LOC_Os10g34010	10	S10_18145180	expressed protein
91	LOC_Os10g34470	10	S10_18387130	HD domain containing protein 2, putative, expressed
92	LOC_Os10g34480	10	S10_18393744	cytochrome P450, putative, expressed
